# Lower speciation, not higher extinction, drove African megaherbivore diversity collapse

**DOI:** 10.1038/s41467-026-76105-2

**Published:** 2026-07-31

**Authors:** Juan L. Cantalapiedra, Ignacio A. Lazagabaster, Fernando Blanco, Torsten Hauffe, Faysal Bibi, María Ríos, Bastien Mennecart, Juha Saarinen, Daniele Silvestro, Manuel Hernández Fernández, Oscar Sanisidro

**Affiliations:** 1 Departamento de Paleobiología, Museo Nacional de Ciencias Naturales, MNCN-CSIC, 28006 Madrid, Spain; 2 Museum für Naturkunde, Leibniz Institute for Evolution and Biodiversity Science, 10115 Berlin, Germany; 3 GloCEE, Departamento de Ciencias de la Vida, Universidad de Alcalá, 28805 Alcalá de Henares, Spain; 4 Centro Nacional de Investigación sobre la Evolución Humana (CENIEH), 09002 Burgos, Spain; 5 Department of Ecology, Evolution & Behaviour, University of Liverpool, CH64 7TE Liverpool, UK; 6 Departamento de Ecología y Evolución, Estación Biológica de Doñana (CSIC), Sevilla, Spain; 7 Gothenburg Global Biodiversity Centre, Department of Biological and Environmental Sciences, University of Gothenburg, 40530 Gothenburg, Sweden; 8 The School of Mathematics and Statistics, The Open University, Turing Building 310, MK7 6AA Milton Keynes, UK; 9 Department of Biology, University of Fribourg and Swiss Institute of Bioinformatics, 1700 Fribourg, Switzerland; 10 Department of Earth Sciences, GeoBioTec, Nova School of Science and Technology, Universidade NOVA de Lisboa, Campus de Caparica, 2829-516 Caparica, Portugal; 11 Museum d’Orléans pour la Biodiversité et l’Environnement, 45000 Orléans, France; 12 Department of Geosciences and Geography, University of Helsinki, FI-00014 Helsinki, Finland; 13 Present Address: Department of Biosystems Science and Engineering, ETH, Zurich, Switzerland; 14 Departamento de Geodinámica, Estratigrafía y Paleontología, Facultad Ciencias Geologicas, Universidad Complutense de Madrid, 28040 Madrid, Spain; 15 Departamento de Geología Sedimentaria y Cambio Medioambiental, Instituto de Geociencias (CSIC, UCM), 28040 Madrid, Spain

**Keywords:** Palaeontology, Palaeoecology, Speciation

## Abstract

Today’s ecosystems are severely depleted in megaherbivores (≥ 1000 kg) and lack many of the ecological functions once provided by these landscape engineers. Whether attributed to humans or climate, prevailing views of megaherbivore decline have focused on size-biased extinction (i.e., higher extinction rates among the largest species) while neglecting the role of speciation rates. To fully contextualize the decline of African megaherbivores, we examine 23 million years (Myrs) of African ungulate diversification using neural network models. Starting around 7.2 million years ago (Ma), African ungulate faunas witnessed overall accelerations in speciation and extinction rates. While speciation plateaued after 3.6 Ma, extinction rates increased sharply at the onset of the Pleistocene (2.58 Ma), leading to widespread diversity losses across herbivore guilds. Yet, we find that extinction rates are intrinsically lower in large-size lineages and in larger species within families. High-crowned dentitions, which last longer under hard, abrasive diets, are also associated with a suppression of extinction rates. Importantly, speciation rates in the largest herbivores are intrinsically low, and in some lineages were suppressed in response to increasing aridification during the last 5 Myrs. Our findings highlight the macroevolutionary complexity behind megaherbivore decline, advancing understanding of biotic crises involving large-bodied taxa.

## Abstract (web summary)

Massive plant-eating mammals, or megaherbivores, once engineered African landscapes but are nearly gone today. This new analysis of their fossil record suggests their decline was driven not only by high extinction but by low speciation rates worsened by long-term aridification.

## Introduction

Extant mammalian communities harbor only a fraction of the megafaunal diversity that once occupied terrestrial ecosystems^1^. The large mammal extinctions that characterized the late Cenozoic resulted in substantial losses in ecosystem functions^2,3^. Within this process, the collapse of the megaherbivore niche (herbivores exceeding one tonne) stands out for both its severity and its disproportionate functional impact, given the central role that these species play in shaping ecosystem structure and dynamics^4,5^. Thus, the global decimation of megafauna extends beyond the loss of large charismatic species, representing one of the most profound ecological disruptions in terrestrial ecosystems over the past 30 million years.

An extensive body of research has focused on megafaunal declines over the last 100 thousand years (kyrs), presenting multidisciplinary evidence that, especially during the last 15 kyrs, technological capabilities and population densities of human groups would have sufficed to drive populations of large herbivores to extinction in some parts of the world^6–9^. In Africa, however, growing evidence suggests that decisive anthropogenic impacts likely occurred only after an already extended decline in terrestrial large herbivore diversity^10^. Megaherbivore local diversity and abundance started decreasing as early as 4 Ma^10,11^, substantially predating the emergence of hominins capable of hunting or meaningfully modifying their environment. Instead, this prolonged process appears to track key readjustments in primary productivity and herbivore biomass allocation^11^, possibly driven by the late Neogene and Quaternary global increase in aridity^4,12^ and the accompanying expansion of open C_4_ grass-dominated habitats^13^ (whose interrelationship and underlying factors remain debated^12–14^).

Whether driven by human pressures or environmental change, the proposed scenarios for megaherbivore decline typically assume that larger-bodied herbivores faced a greater extinction risk. However, in a scenario of long-term faunal reconfiguration, both extinction and speciation should play comparably important roles^15–17^. For example, phases of low speciation rates may have played a primary role in driving some of the largest biotic crises in Earth’s history^18^. Yet, the contribution of speciation rates to the prolonged decline of the largest herbivores has been entirely overlooked^4,10,11,19–21^ and remains unexplored. This is surprising, since ecological and evolutionary theory offer an alternative scenario for the prolonged decline of African megaherbivores. By virtue of their broad-ranging ecology^22^, megaherbivores should experience reduced spatial and temporal stochasticity in resource availability^23,24^, a buffer that likely lowers their extinction risk. Conversely, a larger size is likely linked to a lower potential for speciation. Low population densities and spatial scaling of resources impose a lower limit on megaherbivore species diversity^25,26^. Moreover, megaherbivore’s starvation-recovery dynamics and their reduced densities should limit speciation, as incipient peripheral populations are more vulnerable to demographic collapse before reaching a stable population size^27^.

During periods of environmental forcing, the impact of body mass on speciation and extinction rates should be understood in conjunction with species’ resource preferences^15,28–30^. In herbivores, enhanced dietary flexibility (e.g. via increased molar crown height or hypsodonty^31,32^) is expected to modulate diversification trends, both by impacting species' intrinsic rates of extinction and speciation^33–35^, as well as by expanding carrying capacity through access to novel or expanding ecological zones^29,36,37^. Accordingly, assessing how environmental changes shaped both speciation and extinction across herbivore guilds should offer fundamental insights into the dynamics of African megaherbivore diversity collapse. Yet despite previous investigations of African herbivore diversity^29,38^, an approach that could simultaneously quantify the interactions between diversification, climate change, and ecology has remained out of reach, constrained by the absence of models capable of integrating all these factors and the shifting interplays between them.

Here, we reconstructed diversification trends in 396 species of African ungulates over the last 23 Myr using a vetted dataset of 3327 fossil occurrences and a novel diversification model based on Bayesian neural networks^39^. We estimated changes in speciation and extinction rates as a function of three traits (body mass, tooth crown height, and phylogenetic affinity) and paleoenvironment (using proxies for C_4_ landscapes expansion and aridity), with an emphasis on the mode and timing of megaherbivore decline. Incorporating phylogeny allows us to account for clade-specific effects that can refine correlations between diversification, paleoenvironment, and traits. Importantly, the model also incorporated time as a predictor, capturing temporal dynamics beyond environmental change. It also made it possible to assess how both the magnitude and direction of trait effects on diversification shift over time or in response to changing environmental conditions.

**Fig. 1:**
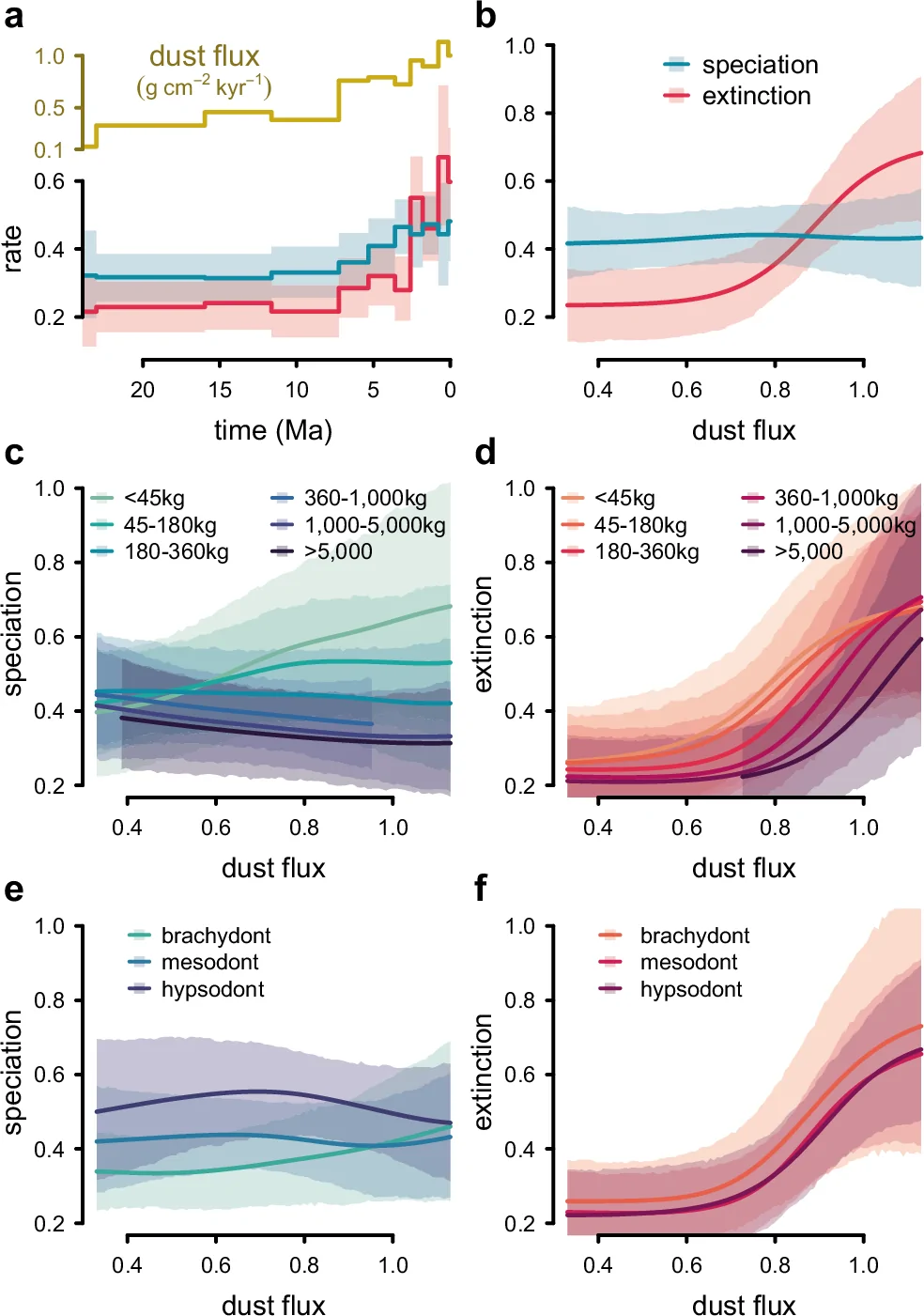
Effects of aridification and traits in diversification. Speciation and extinction rates through time in African ungulates (**A**), with aridification trends at the top^12^. Effect of aridification on diversification rates (**B**). Isolated effect of the interaction of aridification and body mass (**C**, **D**), and hypsodonty (**E**, **F**) on speciation (blueish colors) and extinction (reddish colors). The impact of aridification on speciation was more strongly influenced by body size and hypsodonty, whereas its effect on extinction was more consistent across trait categories. Shaded areas represent the 95% highest posterior density (HPD) from 10,000 posterior samples. All rates are in units of events lineage^-1^ Myr^-1^.

Our analyses reveal the macroevolutionary processes underlying the long-term decline of African megaherbivores, framing this trajectory within a multi-million-year turnover phase in herbivore faunas. From 7.2 Ma, several herbivore lineages underwent an acceleration in both speciation and extinction rates (Fig. 1a). Speciation rates plateaued after 3.6 Ma, while extinction rates tripled from Miocene baselines at the onset of the Pleistocene (2.58 Ma). This sharp extinction phase led to widespread diversity loss across herbivore guilds, marking a severe biotic disruption at the continental scale with ecological and evolutionary implications extending well beyond the disappearance of megaherbivores. Larger-bodied species had lower speciation and extinction rates as predicted by general theory, with dietary flexibility (i.e., greater capacity for grass intake) suppressing extinction and enhancing speciation. Speciation in megaherbivores was further suppressed in some lineages as environmental forcing progressed, predisposing them to severe diversity losses as extinction rates rose across all lineages and ecological strategies in the Pleistocene.

## Results and discussion

**Fig. 2:**
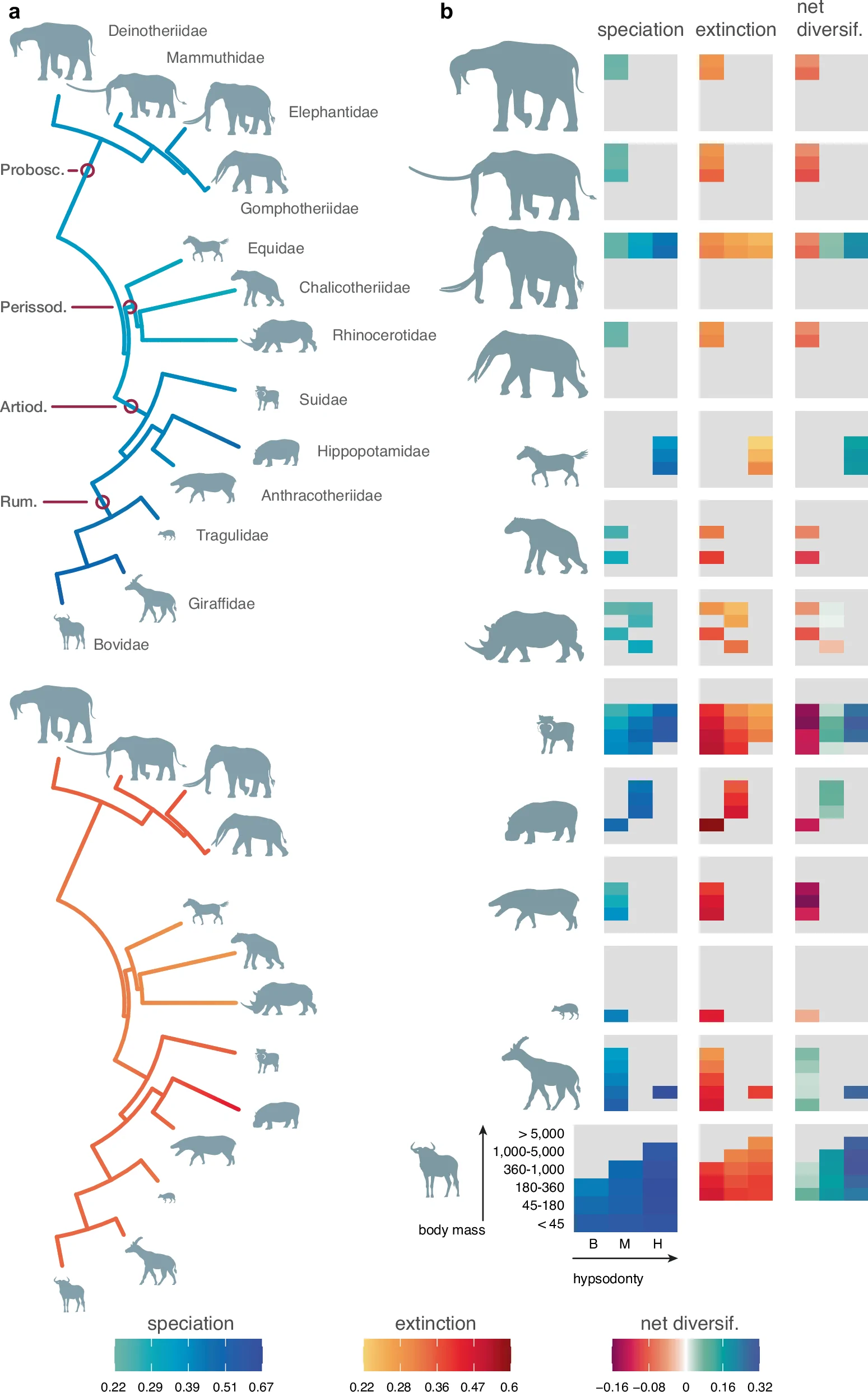
Effect of phylogeny and ecological traits on ungulate diversification. Isolated effect of phylogeny (**A**) in comparison with the combined effect of phylogeny and the ecospace (**B**). The six size categories (in kg) and three hypsodonty categories (B, brachydont; M, mesodont; H, hypsodont) are combined into an ecospace. Note the different scale in positive and negative values of net diversification. Some silhouettes in this and subsequent figures were modified from refs. ^11^ and ^98^, or from ref. ^99^ (used with permission of Asier Larramendi).

**Fig. 3:**
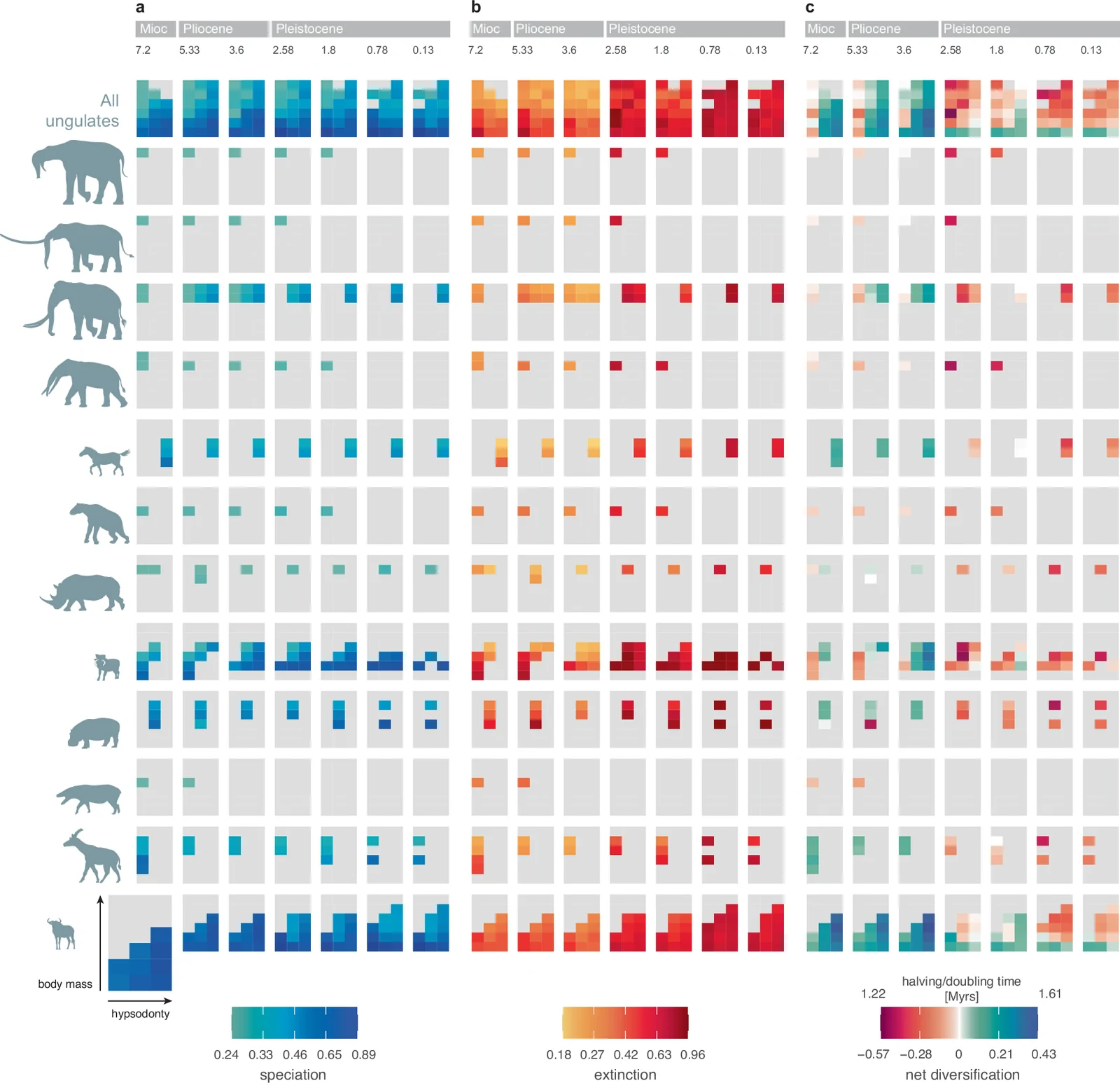
Diversification rates over the last 7 million years, shown by family, body mass, and tooth crown height. Speciation (**A**), extinction (**B**), and net diversification (**C**) rates through time (RTT) are here summarized across time bins, for all African herbivores (top row), as well as by family (same symbols as in Fig. 2) and the ecospace. Numbers at the top indicate the temporal boundaries between bins, in millions of years ago (Ma). Note the different scales for positive and negative net diversification values. For reference, halving and doubling times for diversity are shown relative to diversification rates. Here, RTTs integrate the effects of all the predictors (traits and aridification); predicted RTTs in the absence of aridification are shown in Supplementary Fig. 8. A version of this figure with a complete time series starting 23 Ma is shown in Supplementary Figs. 9–11.

Our interpretation of the macroevolutionary patterns described here is organized around three complementary analytical outputs derived from our Bayesian neural network model (BDNN)^39^. To compare the relative importance of predictors, we report SHAP values, which capture each predictor’s average contribution to species-specific rate variation, and ΔLik, the decrease in model fit when that predictor is permuted across species (Methods; Supplementary Data S1). To describe the direction, magnitude, or shape of a predictor’s effect on rates, we rely on partial dependence (PD) plots (e.g. Figs. 1, 2, S6 and S7). PD plots isolate the effect of one or several focal predictors, making it possible to visualize nonlinear and interactive effects without assuming a fixed functional form^39^. Finally, we generate rates-through-time (RTT) plots that integrate the effects of all predictors simultaneously and reflect the actual inferred dynamics within each subgroup of species defined by their phylogenetic affiliation and ecomorphology (Fig. 3 and Supplementary Figs. 9–11).

### Body mass, environmental change, and diversification

Larger-sized species show lower turnover overall, speciating and going extinct at lower rates than smaller ones (Supplementary Fig. 7). The observed pattern aligns with the larger geographical ranges and wider habitat distributions of large species^22^, which makes them more resilient to spatial resource stochasticity and environmental changes^23,40^. Our findings are consistent with similar trends seen in invertebrates^41,42^, emphasizing that the current extinction threat in larger mammals^43–45^ is not an inherent feature of larger size, and should not be extrapolated over deep time.

The decimation of African megaherbivore diversity resulted primarily from intrinsically low speciation rates and environmentally driven constraints on speciation in the largest lineages, rather than from extinction preferentially targeting larger sizes (Fig. 1). Although long-term aridification and associated environmental shifts explain a threefold increase in extinction rates among African ungulates (Fig. 1b; see Supplementary Data 1), the direction of the effect was relatively uniform across body sizes (Fig. 1d). Even though highest posterior density (HPD) intervals in Fig. 1d are relatively broad, extinction responses to aridification are delayed and attenuated in larger size classes in 88% of the posterior samples (Supplementary Data 1). At peak aridification (maximum extinction risk), species below 45 kg exhibited only slightly higher extinction rates (15%) than those species over 1000 kg (Fig. 1d), but speciated 2.2 times faster (Fig. 1c).

Thus, the negative diversification balance among the largest species is therefore better explained by low or even subdued speciation rates, rather than by particularly elevated extinction rates (Fig. 1c; see further discussion below).

### Phylogeny, hypsodonty, and diversification

Our analyses reveal a complex interaction of phylogeny, body mass, and hypsodonty underlying diversification (Fig. 2). Phylogeny emerged as an important modulator of diversification, especially regarding speciation (Fig. 2, and S3; for speciation: SHAP = 0.090, ΔLik = −38.1; for extinction: SHAP = 0.063, ΔLik = −30.7; see Supplementary Data 1). Here, phylogeny represents evolutionary relationships at the family level and encapsulates inherited traits such as physiology, masticatory functional morphology, biomechanics, social structure, space use, habitat preferences, and dispersal habits. When the effect of phylogeny was isolated (see Methods), artiodactyls showed substantially higher speciation rates, especially in hippopotamids, bovids, and giraffids (Fig. 2a). The highest intrinsic extinction risk was found in hippopotamids, followed by proboscideans and ruminants. Perissodactyls (horses, rhinoceroses, and chalicotheres), on the other hand, were associated with the lowest inherent speciation and extinction rates (Fig. 2a).

Intrinsic diversification potential varied across the phylogeny and among the eighteen ecomorphs (combinations of body mass and hypsodonty categories that approximate functional niches and can be arranged into a two-dimensional ecospace; Fig. 2). For example, body mass seems to have had a limited inherent effect on speciation within proboscidean families (mostly restricted to the two categories above 1000 kg), but size clearly impacted speciation in horses, suids (warthogs and relatives), anthracotheres (an extinct artiodactyl family), and giraffids (Fig. 2b). In contrast, size-selective extinction was more widespread across the tree (body mass × phylogeny: speciation SHAP = 0.087, ΔLik = −15.6; extinction SHAP = 0.084, ΔLik = −19.0; see Supplementary Data S1). Mammutids, horses, chalicotheres, rhinos, hippopotamids, giraffids, and bovids all showed decreasing extinction with increasing size, although the steepness of these gradients varies among groups (Fig. 2b).

The attainment of high-crowned teeth had an overall positive effect on diversification across the phylogeny (see the rightmost column in Fig. 2b; for speciation SHAP = 0.071, ΔLik = −33.8; for extinction SHAP = 0.039, ΔLik = −17.7; Supplementary Data 1; see also the relatively weak interaction effect between hypsodonty and phylogeny; speciation SHAP = 0.078, ΔLik = −7.6; extinction SHAP = 0.085, ΔLik = −12.1; Supplementary Data S1 and Fig. S3). The highest intrinsic diversification rates are observed among hypsodont elephantids, equids, suids, and bovids (Fig. 2b), which suggests that other modifications for an enhanced dental masticatory durability may have also underpinned higher diversification potential in African ungulates [e.g., elongated molars with flat occlusal morphologies that allow for efficient forward shearing forces applied to break down food^36,46^]. Enhanced dental durability in elephantids, suids, and bovids likely improved the survivorship of marginal populations, ultimately promoting the emergence of new species^28,35^, as reflected in their high speciation rates. At the same time, this enhanced viability of isolated groups might also explain milder extinction risk in hypsodont elephantids and suids when compared with their brachydont (low-crowned) and mesodont (moderately high-crowned) counterparts (Fig. 2b; the relationship is less evident in bovids)^33,34,36^.

Taken together, these findings reveal a sorting mechanism at the species level (suppressed extinction and enhanced speciation) by which forms that evolved craniodental modifications for durable and effective processing of nutritionally-poor, more abrasive food became dominant in African ungulate communities.

### Environmental change and ungulate diversification

Abiotic forcing, driven by decreasing atmospheric CO_2_ concentrations and long term global temperature decrease (culminating with the Quaternary glacial cycles), led to long-term aridification and significant restructuring of vegetation in Africa’s terrestrial ecosystems over the last 10 million years^10,47^. These changes are well documented in the African paleoenvironmental record, which integrates diverse sources (e.g., isotopes from paleosols, tooth enamel and leaf wax, pollen, dust deposits, etc) from continental outcrops as well as lacustrine and marine cores^48^. This proxy richness offers insight into both the temporal heterogeneity and the spatial variability of African environments during the late Neogene and Quaternary. However, most paleoenvironmental datasets typically span the last 10 Myrs, and show consistent sampling during the last 5 Myrs^49^. This limits their integration into broader-scale analyses, such as the one undertaken here, which aims to model 23 million years of ungulate diversity dynamics in Africa. We therefore focus on two major aspects of environmental change that are consistently represented across this extended timeframe: increasing aridification [based on rates of dust flux^12^], and the expansion of C_4_ grasses [based on δ^13^C isotopic signal in long chain *n*-alkanes (C_35_)^50^]. Although their interdependence remains incompletely understood, aridity shapes the distribution, spatial variability, and abundance of vegetation types, and both are linked to complex feedbacks involving seasonality, herbivores pressure and fire regimes^51,52^. Both proxies derive from marine cores, and likely reflect aggregated trends at the subcontinental scale. While we interpret them as capturing the dominant environmental transitions across Africa, we acknowledge their spatial averaging and the need for future reconstructions that integrate basin- or region-specific records to resolve intra-continental heterogeneity in a unifying framework, something that current macroevolutionary models do not yet accommodate.

We ran two separate models using each of these proxies (dust flux rates and δ^13^C isotopic signal in long-chain n-alkanes) as the primary environmental predictor, along with a combined model incorporating both variables (see Methods). The model incorporating dust flux as proxy accounted for more variation in speciation and extinction rates across species (Supplementary Figs. 3 and 4; see Methods), yielding a better model than the one based on δ^13^C signal. Despite ongoing debate on the relative timing of the aridity and C_4_ grass expansion in African habitats, both proxies show a marked increase beginning at 7.2 Ma in our time-binned framework (Supplementary Fig. 2). This suggests that differences in the onset timing of arid regimes and C_4_ landscapes are unlikely to explain variation in model fit. Instead, the improvement in fit likely stems from the abrupt rise in dust flux rates at 2.58 Ma, in contrast to the more gradual, sustained increase in the C_4_ grasses indicator since 7.2 Ma. As we will see, the Pliocene-Pleistocene transition, 2.58 Ma, marked a pivotal moment in the diversification dynamics of African herbivores.

We focus the main discussion on the effects of aridification, although the main output from the model based on δ^13^C isotopic signal in long chain n-alkanes (C_35_) is shown in Supplementary Fig. 6. With time explicitly modeled, aridity’s significance reflects more than just arbitrary temporal variation in rates. In other words, the signal of Africa’s aridification explains the timing and magnitude of diversification better than time (effect of dust flux on speciation ΔLik = −31.4; on extinction ΔLik = −79.1; effect of time on speciation ΔLik = −4.7; extinction ΔLik = −8.5; see Supplementary Data and Supplementary Fig. 3). A way to exemplify the impact of environmental change on the diversification of African herbivores is to recalculate speciation and extinction rates after removing its effect (Supplementary Fig. 8). Without the influence of aridification, our model failed to predict net diversity losses across the phylogeny and the ecospace, including the decline among key megaherbivore groups such as proboscideans and hippopotamids (compare Fig. 3 and Supplementary Fig. 8).

Aridification had no net effect on speciation rates when considered in isolation. Its impact emerges only in interaction with the phylogeny and specific ecological contexts or ecomorphs, revealing divergent responses across ecological strategies (Fig. 1c and f, and top row of plots in Fig. 3). Environmental change accelerated speciation rates by around 50% in species under 180 kg, while causing a ~ 20% deceleration in megaherbivores. Despite the broad HPD intervals in Fig. 1c, the response of lineages in different size categories is robust across MCMC iterations. In most iterations (90%), the effect of aridification on taxa below 360 kg differs from larger-bodied taxa. (see Supplementary Data 1). This suppression effect is also modulated by phylogeny (dust flux × phylogeny: SHAP = 0.077, ΔLik = −12.2; see Supplementary Data S1). Giraffids and hippopotamids above one tonne show such deceleration in speciation rates, while proboscideans and rhinocerotids do not (Fig. 3 and Supplementary Fig. 12).

Hypsodonty also modulates the response of speciation to environmental change (Fig. 1e). Despite apparently broad HPD intervals, taxa in different hypsodonty categories show disparate responses to aridification in 91% of the posterior samples (Supplementary Data 1). Moderate levels of aridity correlate with increased speciation rates in hypsodont taxa (Fig. 1e). From 11.6 Ma onwards, environmentally-driven speciation accelerated in hypsodont taxa and peaked between 7.2 and 2.58 Ma, coinciding with an accelerated radiation of hypsodont bovids, suids, and elephantids. However, intensifying aridification in Pleistocene times (after 2.58 Ma) resulted in a slowdown in speciation among hypsodont lineages. This hump-shaped relationship between hypsodonty and environmentally-driven speciation (Fig. 1e) likely reflects three phases. First, the onset and initial expansion of grass-dominated habitats; second, the attainment of optimal productivity conditions and maximal heterogeneity in woody cover and ecotones at intermediate rainfall^52–54^; and third, a general decline in productivity and a reversal to more homogeneous landscapes under extreme values of our aridity proxy.

Aridification is the top predictor of extinction dynamics (SHAP = 0.163, ΔLik = −79.1; Supplementary Data S1), accounting for a threefold increase in extinction rates (Fig. 1b). However, unlike its effect on speciation, this impact was more uniformly distributed across the ecospace, showing less variation with respect to body size or hypsodonty levels (Fig. 1d and f). During the most arid periods, extinction rates are predicted to exceed speciation rates twofold (Fig. 1b). After 2.58 Ma, aridification reached values that predict widespread net diversity losses (Fig. 1a and b). As low-quality grasses expanded at the expense of more productive grasslands and woodlands^55^, primary productivity would have decreased^11^, readjusting the diversity limits of African large herbivore communities^30,56,57^. These developments went hand in hand with increased seasonality and large-scale homogenization of faunas^19^.

The extent to which aridification primarily tracks global physical trends versus ecosystem-level reorganization is still elusive in our understanding of late Cenozoic African environmental dynamics^12,13,51,58^. Our aridity and C_4_ grass proxies likely reflect different mechanistic facets of the same broad transformation of African ecosystems since the Middle Miocene, spanning shifts in seasonality and hydrological regimes to cascading ecological feedbacks involving vegetation structure and fire regimes. Because herbivory both responds to and modulates these trends^59^, faunal dynamics are themselves embedded within the environmental feedback system. In this regard, our results suggest that herbivore turnover is more tightly coupled to the dimension of environmental change captured by moisture availability than to shifts in vegetation composition.

That disentangling these coupled processes is far from straightforward is illustrated by the conflicting biotic responses reported in the literature. In some instances, isotopic values from tooth enamel show lineages adjusting their diets to environmental changes^60–63^. In others, a decoupling of biotic response from environmental change is exemplified by lineages showing dietary conservatism, or in limited changes in the relative local abundance of grazing and browsing taxa^51,61,64^.

A key distinction of our approach is its scale and its emphasis on variation of speciation and extinction rates across lineages and ecomorphs. At this macroevolutionary scale, dietary trends can emerge from differential extinction and speciation rates among lineages, even under widespread conservatism^28,65^. Through such a process of species sorting, major reconfigurations of African ungulate faunas can take place even under the strongest assumptions of dietary conservatism within ungulate genera, tribes, or families.

Future developments in macroevolutionary modeling, such as the framework advanced here, may help reconcile environmental signals operating at basin and regional scales within a unified analytical architecture. Integrating multiple environmental trends into coherent mechanistic models could substantially improve our ability to resolve the spatial heterogeneity of biotic responses across subcontinental regions. Continued refinement of environmental proxies, together with expanded temporal and geographic coverage, will be essential for advancing these integrative approaches and for further clarifying how climatic forcing translated into the uneven macroevolutionary trajectories observed across African ungulate lineages over space and time.

### The restructuring of African ungulate faunas

The earliest signals of environmentally driven restructuring of African faunas emerged in the latest Miocene (our 7.2 to 5.33 Ma bin; Fig. 3, and Supplementary Figs. 9–11). During this period, speciation rates increased in suids, equids below 180 kg, and particularly among hypsodont bovid tribes^29^, in which species weighing over 180 kg were recorded for the first time. In bovids and equids, these elevated rates of species proliferation encapsulate dispersal of only three species from Eurasia (in three different genera; Supplementary Fig. 13), suggesting that the diversification signal is not substantially confounded by the influx of immigrant taxa. In contrast, the addition of brachydont large-sized species had already ceased in some lineages. The last speciation among anthracotheres and the last dispersal of a deinothere into Africa had occurred around 10 Ma. Other brachydont megaherbivores would soon follow this pattern, indicating the importance of speciation potential and feeding strategy for the decline of the largest African ungulates. The latest Miocene and early Pliocene (7.2–3.6 Ma) witnessed the last speciation and dispersal events among the browsing gomphotheres and mammutids, with a noticeable drop in speciation among other large-size families starting around 5 Ma (Supplementary Figs. 12 and 13). Overall, Pliocene extinction rates remained at moderate levels (with the exception of small brachydont suids and smaller hippopotamids). Even in the absence of elevated extinction among the largest herbivores, parts of the megaherbivore adaptive zone had begun to empty well before the Pleistocene due to their low speciation rates.

However, some megaherbivore ecomorphs would still exhibit net diversity gains (Fig. 3, and Supplementary Figs. 9–11). Between 5.33 and 3.6 Ma, the radiation of elephantids accelerated, leading to the emergence of the first hypsodont multi-ton herbivores^28,36^ (Fig. 3). This development, along with increased speciation in bovids and suids (and suppressed extinction rates in bovids, as well as mesodont and hypsodont suids and elephantids; Fig. 3), solidified the dominance of savannah faunas in Africa. We suggest that the magnitude of this radiation (with the diversity of some ecomorphs doubling every 1.6 Myrs; Fig. 3) stems from several combined processes. The expanding savannah biome provided access to a much enlarged ecological zone^29,36,37^, and promoted habitat heterogeneity, increasing the carrying capacity of the whole system^16^. This effect was multiplied by the higher intrinsic speciation and lower extinction rates of species capable of mixed feeding or grazing (typically meso- and hypsodont taxa; Fig. 1 and Supplementary Fig. 7). By allowing the incorporation of lower quality forage, dietary flexibility increases population persistence, thereby reducing extinction risk^33,34^ and enhancing the survival of diverging isolates, which promotes speciation^35^. The emergence of novel dietary and social strategies would have relaxed the crowding effect, promoting the coexistence of species in sympatry^28,66^. These innovations included the emergence of migratory habits, which would have ensured resource access by allowing feeding across multiple seasonal landscapes^24^, and of specialized grazers, capable of partitioning roughage types^67^. The combination of these two non-exclusive traits would have promoted speciation through the isolation of populations under local adaptive pressures^68^.

Speciation rates in browse-restricted species moderately increased after 7 Ma, likely driven by intensification of forest patches fragmentation^69,70^. However, they underwent an even larger increase in their extinction rate (Fig. 3, and Supplementary Fig. 10). Browsers above 45 kg experienced sustained net diversity losses ever since, which could be explained by the reduction in total area of suitable habitat and the resulting intensification of the crowding effect^16^, which substantially reduced the carrying capacity of browse-specialists in the African species pool. Nevertheless, and despite diversity losses among brachydont taxa, the Late Miocene and Pliocene constituted a diversification bonanza for ungulate faunas, with net positive diversification across most regions of ecospace (i.e., net diversity gains; Fig. 3 and Supplementary Fig. 11).

In contrast, the Pleistocene marked a transition to negative diversification across families and ecologies, with speciation rates remaining at Pliocene levels while extinction rates doubled (Fig. 1a). This extinction phase likely represents one of the most impactful biotic crises in Cenozoic times. The early Pleistocene saw an intensification of the high-latitude glacial cycles, which drove profound changes in African climate^71^ as reflected by a marked increase in our aridification proxy 2.58 Ma (Supplementary Fig. 2). Global temperature fluctuations, increased seasonality in precipitation regimes, and suppressed CO_2_ atmospheric concentrations led to substantial ecological change in African ecosystems^13^. This new phase of abiotic forcing also triggered distinct speciation and extinction patterns across lineages and ecomorphs, ultimately shaping the transition from the Miocene–Pliocene phase of elevated diversification to present-day depauperate ecosystems.

Between 2.58 and 0.78 Ma, speciation rates in bovids decelerated, and extinction rates remained moderate in bovids, giraffids, and horses (Fig. 3). Bovids were the only family to have maintained overall net positive diversification through the Early Pleistocene (Fig. 3c). Suids, hippopotamids, mammutids, gomphotheres, and the sole surviving species of deinothere experienced marked acceleration in extinction rates. However, similar to horses and ruminants, large ungulates like elephantids, chalicotheres, and rhinos showed moderate rates of extinction compared to other groups, emphasizing that increased extinction risk was not a feature of megaherbivore families, or in large-size ecomorphs within families.

Starting in the Middle Pleistocene (0.78 Ma, onwards), speciation rates increased in smaller suids and bovids, including browsing forms (Fig. 3a). This could reflect both a late radiation of lineages in small woodland patches^72^ or the replacement of grazing guilds by smaller browsers and mixed feeders in very dry environments^73^. From this point onward, extinction rates increased across the ecospace and the phylogeny. This trend was delayed in horses and bovids, but they ultimately joined other groups in an indiscriminate pattern of extinction over the last 130 kyrs. There is no evidence of size-selective extinction during this final phase of collapse (Fig. 3b).

As habitats became more seasonal and less productive^12^, seasonally variable and patchily distributed resources could no longer support the abundance and diversity of wide-ranging multi-ton grazers (Supplementary Figs. 9–11). As megaherbivore faunas became more similar across regions (reflecting a decline in provincialism^19^), continental diversity declined, likely contributing to more homogeneous and less diverse vegetational landscapes^59^. As the carrying capacity of ecosystems shrank, extinction rates increased across all clades, and only in bovids did they remain low enough to sustain increases in diversity for a longer period. This suggests a crucial role for bovid-specific features (such as facultative drinking, foregut fermentation, a variety of social systems, etc.) in extracting energy from changeable environments.

The macroevolutionary processes described here must be interpreted within the spatial and environmental limits of the African fossil record^13^ (Supplementary Fig. 14). Neogene and Quaternary African sites primarily capture a broad range of savanna environments, including woodlands, wetlands, and grasslands. It is mainly within this environmental and spatial context that patterns of African megaherbivore decline had been previously documented^10,11,19^, and within which our findings should be understood. The processes described here are not straightforwardly extrapolated to tropical forests^74^, where brachydont taxa likely were buffered against extinction, and where grazers did not reach such high levels of diversity^70,73^.

Previous studies on continental and regional megafaunal depletion centered primarily on extinction patterns (particularly during the Late Quaternary, the last 130 kyrs), while largely overlooking the role of speciation rates. By analyzing all African ungulates across the entire Neogene-Quaternary, we provide a wider context for the complex mechanisms that drove the long-term loss of African megaherbivores. Our results show that both the timing and magnitude of this decline closely track a prolonged aridification trend and associated environmental changes. Specifically, the loss of large species and the shift in biomass towards medium- and small-sized herbivores resulted from low, and even suppressed, speciation rates among large-bodied lineages, rather than elevated extinction rates. This scenario contrasts with notions of an abrupt megaherbivore decline attributed primarily to Late Pleistocene extinctions, which, as we show, lacked size or dietary selectivity. While other drivers (e.g., dispersal of newcomers, predation by humans) could have contributed to some extinction events, they were not the cause of the widespread loss of entire ecological niches. Instead, it was the net balance of extinction and speciation rates over geological time, driven by extrinsic environmental changes, that fundamentally restructured African herbivore communities, producing the severely depleted ecosystems of today.

## Methods

### Occurrence and ecomorphological data

Occurrence and trait data came from diverse publications^28,75,76^, which in turn were based on data from the NOW database (https://nowdatabase.org/now/database/)^77^, the Paleobiology Database (PBDB; https://paleobiodb.org), and extensive literature surveys (see references in the referred studies). In these articles the pipeline included a filtering and improvement of taxonomic and temporal information of the paleontological records^28^. Although our analyses mainly focus on the Neogene and Quaternary (the last 23 Myrs), we truncated our occurrence data at 30 Ma to avoid artifacts (e.g., inflated speciation at the beginning of the time series). From the remaining dataset, we excluded families with a very low number of occurrences and/or species (Barytheriidae, Gelocidae, Mixtotheriidae, Moeritheriidae, Palaeomastodontidae, Camelidae, Cervidae).

Hypsodonty information is coded as a three-state trait (brachydont, mesodont and hypsodont)^78,79^ derived from the NOW Database and expert opinions. Body mass was coded using the nine categories defined by Andrews et al.^80^. These were then reconfigured in order to more closely fit a logarithmic series. The categories below 45 kg were grouped under a single category; 45–90 kg and 90–180 kg were also combined; category 180–360 kg was kept; and the category above 360 kg was subdivided in the categories 360–1000 kg, 1000–5000 kg and above 5000 kg. The resulting states are defined by the mass thresholds 45 - 180 - 360 - 1000 - 5000 kg. This new categorization fills several purposes. First, each state is represented by a comparable number of species, enabling a robust comparison among different ecomorphological types. Second, it provides more detailed patterns of diversification above the 1000 kg threshold (the megaherbivore niche). Finally, the new configuration is more similar to logarithmic-defined mass bins^11^, than the original classification, ensuring that we are comparing size categories through which a more comparable amount of energy enters the system (as predicted by the energetic equivalence hypothesis)^24,81^. Importantly, the birth-death model based on neural networks (BDNN) used here is not a joint model of trait evolution and species diversification, which relaxes the requirement for a species-level phylogeny^82^. Consequently, continuous traits do not need to strictly conform to logarithmically defined bins to avoid violating the assumptions of a trait evolution submodel.

### Phylogeny

To account for the effect of shared evolutionary history on diversification trends, we compiled a phylogenetic tree of all African ungulates at the family level based on several sources^83–87^. To ensure that our diversification model could have enough data at each tip to correctly parametrize the tree-mediated correlations with speciation and extinction, we fused some small families under a larger clade. Basal giraffoids, giraffids, prolibytherids, and climacoceratids were assigned to the tip ‘Giraffidae’. The only stegodontid species was included under the ‘Elephantidae’ tip. The phylogeny is intended to represent evolutionary distances between the emergence of each of the families and their defining features. Thus, the phylogeny was calibrated so that the tips would end at the oldest record of each family [not necessarily in Africa and obtained from Blanco et al.^76^]. To calibrate the phylogeny we used the function *timePaleoPhy* in the *paleotree* R library^88^ (with arguments type = "mbl", vartime=0.5, dateTreatment = "firstLast", add.term=FALSE).

Our database consists of 3327 occurrences of 396 species, a 13-tip family tree and body mass and hypsodonty information for the 396 species. Across the family tree the diversity is as follows: Deinotheriidae (3 spp), Mammutidae (5 spp), ‘Elephantidae’ (+ Stegodontidae; 19 spp), Gomphotheriidae (14 spp), Equidae (19 spp), Chalicotheriidae (4 spp), Rhinocerotidae (24 spp), Suidae (54 spp), Hippopotamidae (21 spp), Anthracotheriidae (15 spp), Tragulidae (6 spp), ‘Giraffidae’ (+ Prolibytheridae and Climacotheratiidae; 27 spp), and Bovidae (185 spp).

To include our 13-tips family-level phylogeny in the diversification model, we had to transform it into continuous variables. Using eigenvector decomposition in the *PVP* R package^89,90^, we obtained three vectors that captured 73% of the phylogenetic information encapsulated in the tree (Supplementary Fig. 1). These three variables were combined with hypsodonty and body mass in a single dataframe that was incorporated into the analysis.

### Environmental proxies

The environmental and climatic history of Africa during the Neogene and Quaternary is well documented, enabling the use of regional proxies to capture distinct aspects of long-term environmental trends. Accordingly, we prioritized regional proxies over global environmental datasets [e.g. global paleotemperature or concentrations in atmospheric CO_2_^14,91^]. The scenario of a protracted decline of megaherbivores has been linked to the spread of C_4_ grasslands in the continent^10^. This environmental transformation has two potential facets highly relevant to herbivores. The first involves changes in landscape structure and in the physiognomy and nutritional quality of forage plants. C_4_ grass are less nutritious and contain higher abrasive particles, and thus are expected to have an impact on dental wear, foraging behavior^92^, and the energetics of primary consumers^11^. A second aspect of this transformation is the associated aridification. Although the timing and interplay between increasing aridity and grass expansion remain debated^12^, aridification likely has a closer relationship to productivity, potentially constraining regional diversity and influencing fluctuations in biomass and lineage-level abundance^11,56^.

We incorporated these two facets into separate diversification models, treating aridity and C_4_ grass expansion as time-dependent environmental variables. We used dust deposition rates in deep-sea drill cores as a proxy for aridification^12^, and δ^13^C in long chain *n*-alkanes (C_35_) as a precise signal for C_4_ grasses expansion^50^. Dust flux data was retrieved from cores off Northwest Africa, while *n*-alkanes coresites are located in the Somali Basin and the Red Sea.

Our diversification model allows for the inclusion of more than one time-dependent curve. However, when environmental curves are highly correlated (like here; Kendall correlation on our binned data, *P* = 0.0003, *τ* = 0.782) it may be difficult for the model to find a reliable solution^39^. Nevertheless, a model where both proxies were included yields the same conclusion: aridity has a larger explanatory power than C_4_ grass expansion (Supplementary Figs. 3–5). In particular, aridity explains almost a threefold increase in extinction rates (Fig. 1 and Supplementary Fig. 3). The expansion of C_4_ grasses accounts for only a twofold increase (Supplementary Figs. 4 and 6).

### BDNN PyRate

To model the complex effect of our predictors (body size, hypsodonty, phylogeny and paleoenvironment) in a cohesive framework, we used the BDNN model as implemented in the software *PyRate* [^39^; https://github.com/dsilvestro/PyRate]. BDNN is a birth-death diversification model that can accommodate multiple time and trait-mediated effects and their interactions. The model makes use of the flexibility of neural networks so that these effects are not limited to simple monotonic functions.

The BDNN model allows time to be a predictor itself following a temporal binning system. Since increasing the number of temporal bins increases the computational complexity of the model exponentially, we opt for bins based on geological divisions (rather than, for example, 1 Myr bins). Our binning system is as follows: Oligocene (before 23.03 Ma), Early Miocene (Aquitanian + Burdigalian, up to 15.97 Ma), Middle Miocene (Langhian + Serravallian, up to 11.63 Ma), Tortonian (up to 7.2 Ma), Messinian (up to 5.33), Early Pliocene (Zanclean, up to 3.6 Ma), Late Pliocene (Piacenzian, up to 2.58 Ma), Gelasian (up to 1.8 Ma), Calabrian (up to 780 Kya), Middle Pleistocene (Chibanian, up to 129 Kya), and Late Pleistocene and Holocene (Chibanian, up to the present day). A total of 11 bins. This temporal framework enhances modeling resolution where data is most abundant, applicable to both occurrence records and the dust flux dataset.

BDNN implements a birth-death process based on occurrence data. At the same time, it allows preservation and sampling rates to shift over time and across lineages. The temporal sampling shifts need to be defined a priori, and we used the same binning scheme as described, but accounting for one extra shift in the Pleistocene-Holocene boundary, 11.7 Kya.

Following the binning scheme defined for temporal rate shifts, BDNN automatically computes bin-averaged values for all time-varying variables. To ensure full control and robust results in the diversification analyses, we first binned the raw data and then applied z-transformation to all continuous environmental variables prior to model computation (see Supplementary Data 1).

BDNN analyses were run for 50 million generations over 10 replicates of the occurrence dataset, where record ages were resampled among their temporal ranges. The MCMC chain was sampled every 5000th generation. Example commands to replicate our *PyRate* BDNN analyses are provided in the BDNN Commands section. We discarded the initial 10% of the iterations as burn-in, and extracted 1000 random samples from each run for a combined total 10,000 samples.

As a sensitivity analyses, we run a version of the analyses using the African Land Mammal Ages^93^ plus the Late Pleistocene, using one replicate of the data. We compared the posterior probability of both runs: the version with global geological divisions has a posterior of −5525.7 (95 HPD −5614.8, −5439.4); the version with African Land Mammal Ages has a posterior of −5829.2 (95 HPD −5912.1, −5757.7), a substantial drop in the fit.

In continental-scale assessments of diversity and diversification processes, extinction and speciation dynamics inherently include regional extirpation and the immigration of taxa originating elsewhere. To account for the role of dispersal in shaping speciation rates among African ungulates, we estimated first appearance dates (FADs) of species and genera both in Africa and in Eurasia. Then we compared the ages of these FADs, and considered that a taxon had an Eurasian origin if the Eurasian FAD was at least 0.2 Myr older than the African LAD. (Supplementary Fig. 13). The proportion of FADs attributed to Eurasian-origin genera peaks during 11.6–7.2 Ma. The proportion of FADs of species with an Eurasian origin is substantially lower and peaked in the 7.2 - 5.33 Ma bin. Instances where the speciation pattern of a given family is underpinned by dispersal events into Africa have been mentioned in the main text.

### Assessing predictor importance

Neural networks do not directly provide measures of predictor importance. We assessed the impact of the factors on African ungulate diversification dynamics through the implementation of algorithms adapted from explainable artificial intelligence (xAI)^39^. In particular, we chose SHAP values [SHapley Additive exPlanation^94^] and feature permutation^95^ to assess the relative importance of traits, phylogeny, and environmental predictors on diversification.

SHAP values are indicative of the influence of each predictor and their interaction on each species-specific rate^39^ by quantifying how much rates differ from the average rate across species due to the predictor value. A second approach is to estimate the decreases in the likelihood, or ΔLik, after shuffling the values of the focus predictor (Supplementary Figs. 1, 3–5). This method enables the simultaneous permutation of all values, analogous to shuffling taxon labels across a phylogeny, a commonly employed technique in phylogenetic comparative methods. An interaction between two predictors is deemed significant if the reduction in model fit (resulting from simultaneously permuting both predictors) exceeds the combined reductions observed when each predictor is permuted individually^39^.

### Visualizing predictor effects

To visualize the effect of a given variable or pair of variables over diversification rates we used partial dependence (PD) plots^39,96^. PD plots isolate the effect of the focal predictors by marginalizing over the other variables. This is achieved by calculating averaging rates across the range of the focal predictor while keeping the remaining variables at their observed values. For all predictors varying over time, we used their values at the times of the species’ origination and extinction, respectively. PD plots also allow us to visualize the credible intervals by calculating the effects across mcmc samples (e.g. Fig. 1). *PyRate* BDNN automatically generates many PD plots using the -plotBDNN_effects command as implemented in the *PyRate* script, but this is so far limited to the effect of one or two variables on a rate.

To isolate the combined effect resulting from more than two predictors, the user can run the -BDNN_interaction command. This is the case for the effect of phylogeny, which is here reconstructed from the effect of the three phylogenetic eigenvectors (Fig. 2a). To generate more complex plots showing the interaction of phylogeny and our two traits (i.e., Figs. 2b, 3, and Supplementary Figs. 8–11), we used heatmaps as implemented in the *ggplot2* R library^97^.

We used two approaches for exploring complex interactions of predictors with diversification. First, from data generated using the -BDNN_interaction, as explained above. This implementation allows the user to extract PD rates for a given combination of predictors. This means, we get the expected rates at the inferred times of species’ origination and extinction and display in the cells of the heatmaps the average rate of all species either emerging or going extinct in the respective time bin but not for the moments in time where a species is alive. Although not used here, the model could predict unobserved predictor combinations, and ask, for example, what would be the extinction risk of, let’s say, a 45 kg, hypsodont deinothere during the Late Pleistocene. This differs from more traditional rates through time (RTT) plots where inferred species-time-specific rates based on all predictors are averaged across all species being extant at the respective moment in time. These are called marginal rates because they are calculated for the subset of species being extant at a given moment in time, but should not be confused with marginalizing over a subset of predictors to obtain the PD rates.

Second, we implemented PD-derived rates through time (PDRRT) as a new visualization method in the *PyRate* BDNN library. For this, we marginalize over predictor values over time rather than only at the origination and extinction events (as we would when calculating the PD rates above). This method is computationally demanding but allows us to assess diversification dynamics through time, integrating out the effect of any number of predictors while all focal predictors retain their influence. For example, we can examine how RTTs for African ungulates would appear in the absence of aridification, as shown in Supplementary Fig. 8.

In summary, it is important to differentiate the three ways in which we have visualized the patterns and effects derived from our BDNN model:

1. **RTT plots** (Fig. 1a) are marginal rate estimations that are obtained as the species- and time-specific rates outputted by the neural networks, thus integrating the effects of all the predictors [command -plotBDNN]. We can also obtain RTT for subsets of species, as done for Fig. 3, and Figs. S9 to S11. [command -plotBDNN and -plotBDNN_groups./RTT_groups.txt].
2. **PD plots** (Fig. 1b–f, 2, Figs. S6b–f, and S7) to isolate the effect of one or several predictors, giving the influence of these variables at the origination and extinction events. [command -plotBDNN_effects].
3. **PD rates through time plots** (PDRTT; Fig. S8), combining the former two to display hypothetical diversification dynamics over time, allowing for the effect of one or more predictors to be dismissed. [command -BDNN_PDRTT and -plotBDNN_groups./RTT_groups.txt].

### BDNN commands

We provide detailed commands for the *PyRate* BDNN pipeline, including running the main analysis and computing the effects, importance, rates through time, and customized among-predictor interactions. All this can be found in Supplementary Notes. See further information of the arguments in https://github.com/dsilvestro/PyRate/blob/master/tutorials/pyrate_tutorial_bdnn.md.

### Reporting summary

Further information on research design is available in the Nature Portfolio Reporting Summary linked to this article.

## Supplementary information

Supplementary Information

Transparent Peer Review file

Reporting Summary

Description of Additional Supplementary Files

Supplementary Data 1

## Source data

Source data

## Data Availability

Trait, phylogenetic, and occurrence data are deposited at https://doi.org/10.6084/m9.figshare.29589368. Source data are provided with this paper.

## Code availability

Code for reproducing *PyRate* analyses is available in Supplementary Notes 1 and at https://github.com/dsilvestro/PyRate. Code for generating heatmap figures is available at https://doi.org/10.6084/m9.figshare.29589368.
