# Supplementary material for: Lower speciation, not higher extinction, drove African megaherbivore diversity collapse
Source: Nat Commun. 2026 Jul 31;17:9373. doi: 10.1038/s41467-026-76105-2 (PMC13534676; doi:10.1038/s41467-026-76105-2)

Supplementary Information

Lower speciation, not higher extinction, drove African megaherbivore diversity collapse

Juan L. Cantalapiedra, Ignacio A. Lazagabaster, Fernando Blanco, Torsten Hauffe, Faysal Bibi[2], María Ríos, Bastien Mennecart, Juha Saarinen, Daniele Silvestro, Manuel Hernández Fernández & Oscar Sanisidro

Supplementary Notes

The *PyRate* BDNN pipeline was run using the following commands.

Run BDNN

```
python3 ./PyRate.py occs.africa.Mass.Hyp.Horiz.bdnn_PyRate.py \
-n 50000000 \
-s 5000 \
-p 5000 \
-j 1 \
-mG \
-pP 1.5 0 \
-qShift sampling_times_BDNN.Africa.txt \
-trait_file body.mass.45.180.360.1000.5000+Phylo.73Perc.3dims+Hyp.data.txt
\
-fixShift sampling_times_BDNN.Africa.txt \
-BDNNmodel 1 \
-BDNNnodes 20 10 \
-BDNNtimevar dustFlux.loess.scaled.txt \
-edgeShift 23.03 0 \
-out _dustFlux.loess
```

Compute Effects [produces PDP plots]

```
python3 ./PyRate.py \
-plotBDNN_effects ./Combined_mcmc.log \
-b 0 \
-thread 10 0 \
-resample 1000 \
-plotBDNN_transf_features ./dustFlux.loess.mean.SD.txt \
-root_plot 23.03
```

Compute Importance of predictors (while grouping the effect of phylogeny)

```
python3 ./PyRate.py \
-BDNN_pred_importance ./Combined_mcmc.log
-resample 1000 \
-b 0 \
-thread 10 0 \
```

```
-BDNN_pred_importance_nperm 100 \
-BDNN_groups '{"phylogeny": ["phylo.1", "phylo.2", "phylo.3"]}' \
-root_plot 23.03
```

Compute Importance with interactions (while grouping the effect of phylogeny)

```
python3 ./PyRate.py \
-BDNN_pred_importance ./Combined_mcmc.log \
-resample 1000 \
-b 0 \
-thread 10 0 \
-BDNN_pred_importance_nperm 100 \
-BDNN_groups '{"phylogeny": ["phylo.1", "phylo.2", "phylo.3"]}' \
-BDNN_pred_importance_interaction \
-root_plot 23.03
```

Compute PD rates for combinations of predictors [e.g. Fig. 2]

```
python3 ./PyRate.py \
-BDNN_interaction ./Combined_mcmc.log \
-BDNN_interaction_fix \
-BDNN_groups '{"phylo.1": ["phylo.1"], "phylo.2": ["phylo.2"], "phylo.3":
["phylo.3"], "body.mass.andrews.6": ["body.mass.andrews.6"], "hypsodonty":
["hypsodonty"]}' \
-b 0 \
-thread 10 0 \
-resample 10000 \
-root_plot 23.03
```

Estimate rates-through-time

```
python3 ./PyRate.py \
-plotBDNN ./Combined_mcmc.log \
-b 0 \
-resample 10000
```

Estimate rates-through-time for subsets

```
python3 ./PyRate.py \
-plotBDNN ./Combined_mcmc.log \
-b 0 \
-resample 10000 \
-plotBDNN_groups ./RTT_groups.txt
```

Estimate partial dependence rates-through-time

```
python3 ./PyRate.py \
-BDNN_PDRTT ./Combined_mcmc.log \
-BDNN_groups '{"phylogeny":["phylo.1", "phylo.2", "phylo.3"], "time":
["time"], "body.mass.andrews.6": ["body.mass.andrews.6"], "hypsodonty":
["hypsodonty"]}' \
-b 0 \
-resample 10000 \
-plotBDNN_groups ./RTT_groups.txt
```

Supplementary Figures

Supplementary Figure 1. Phylogenetic eigenvectors. The three phylogenetic eigenvectors obtained from the calibrated phylogeny of African families mapped on the tree.

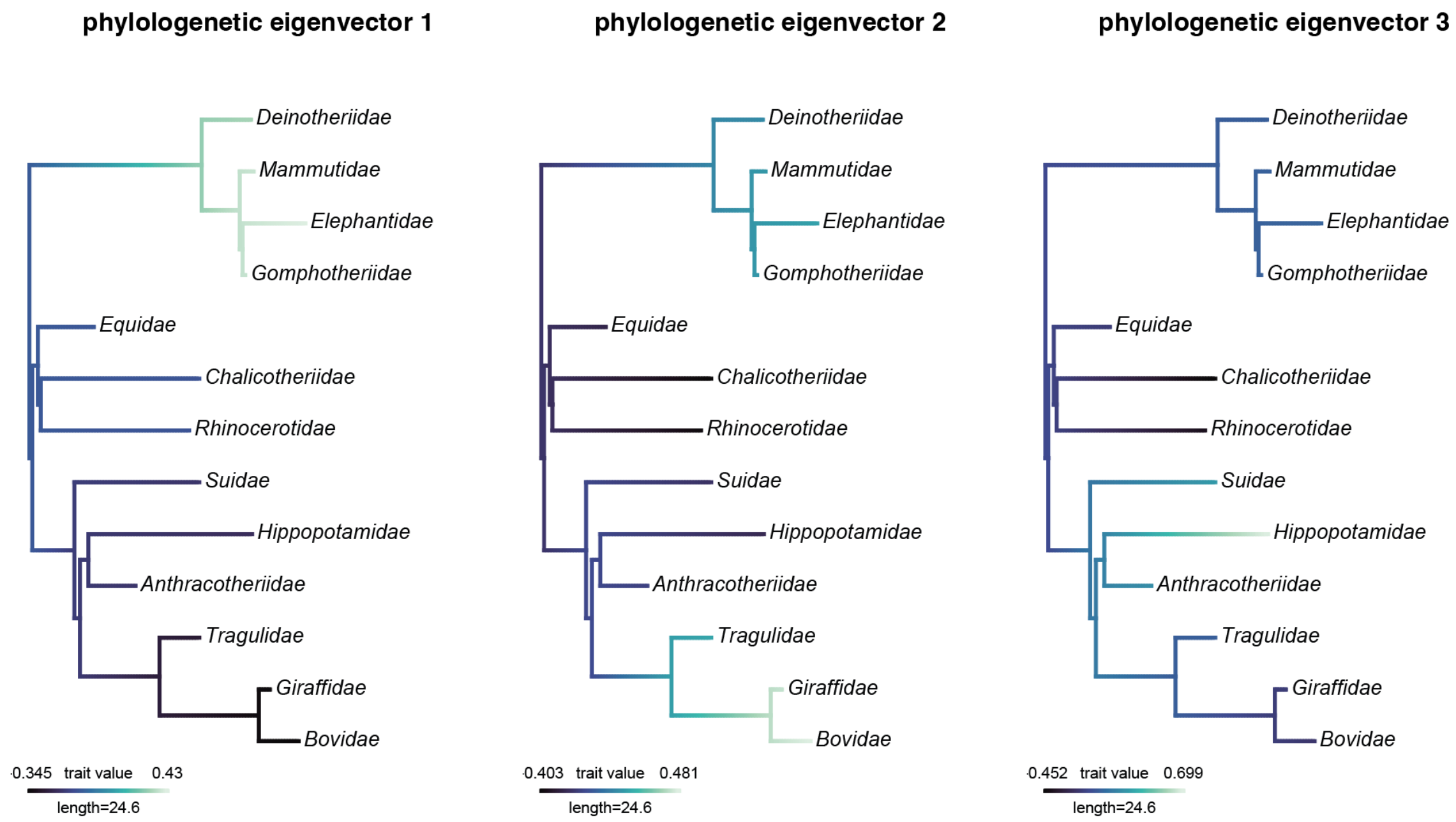

Supplementary Figure 2. The two environmental proxies explored are here plotted through time for comparison. Their values have all been scaled from 0 to 1.

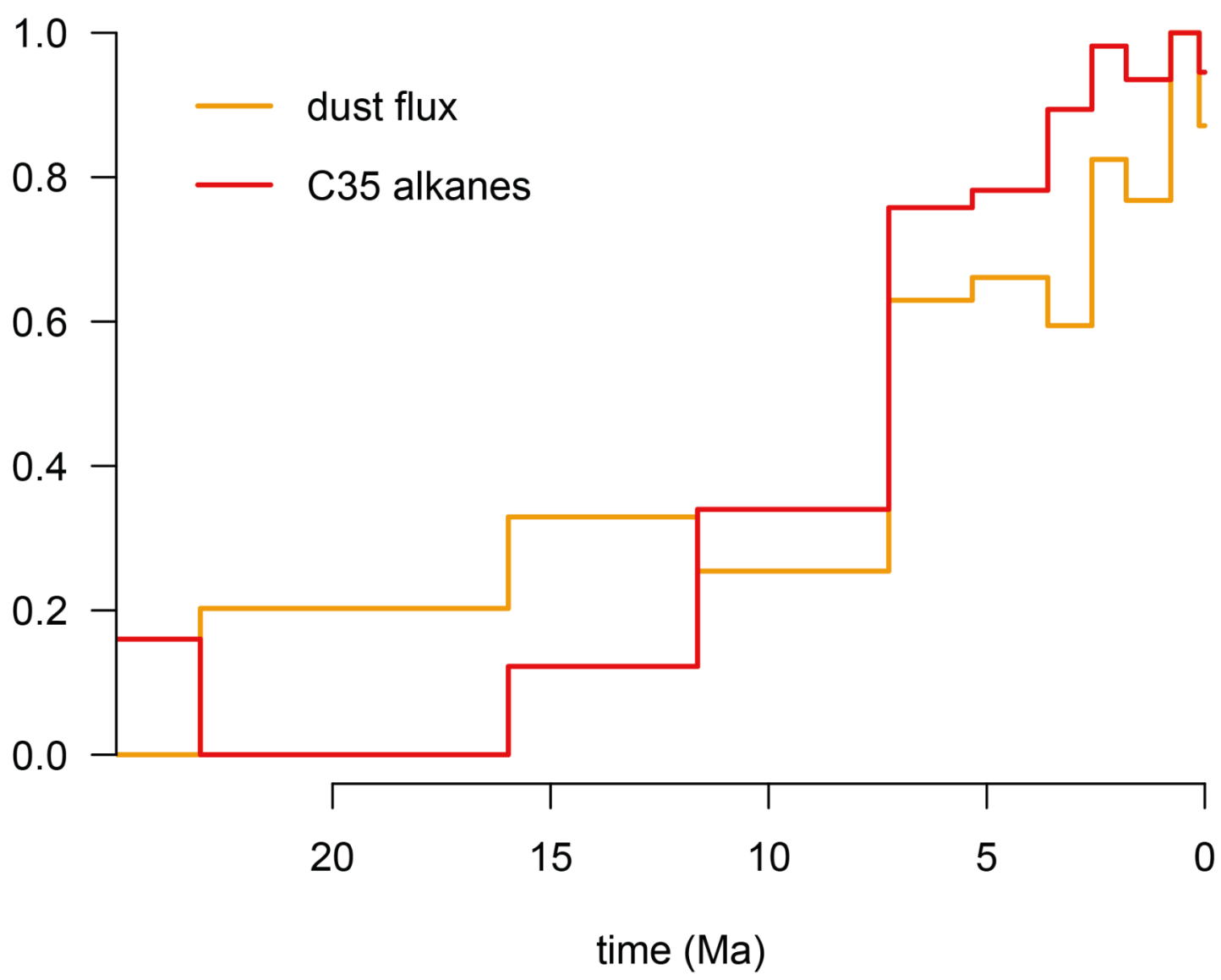

Supplementary Figure 3. Predictors importance of our model with dust flux as environmental proxy. Importance is measured as the decrease in the model fit ΔLik (after shuffling the values of the focus predictor; **A**) and the contribution to the rate value by each predictor (as SHAP values; **B**).

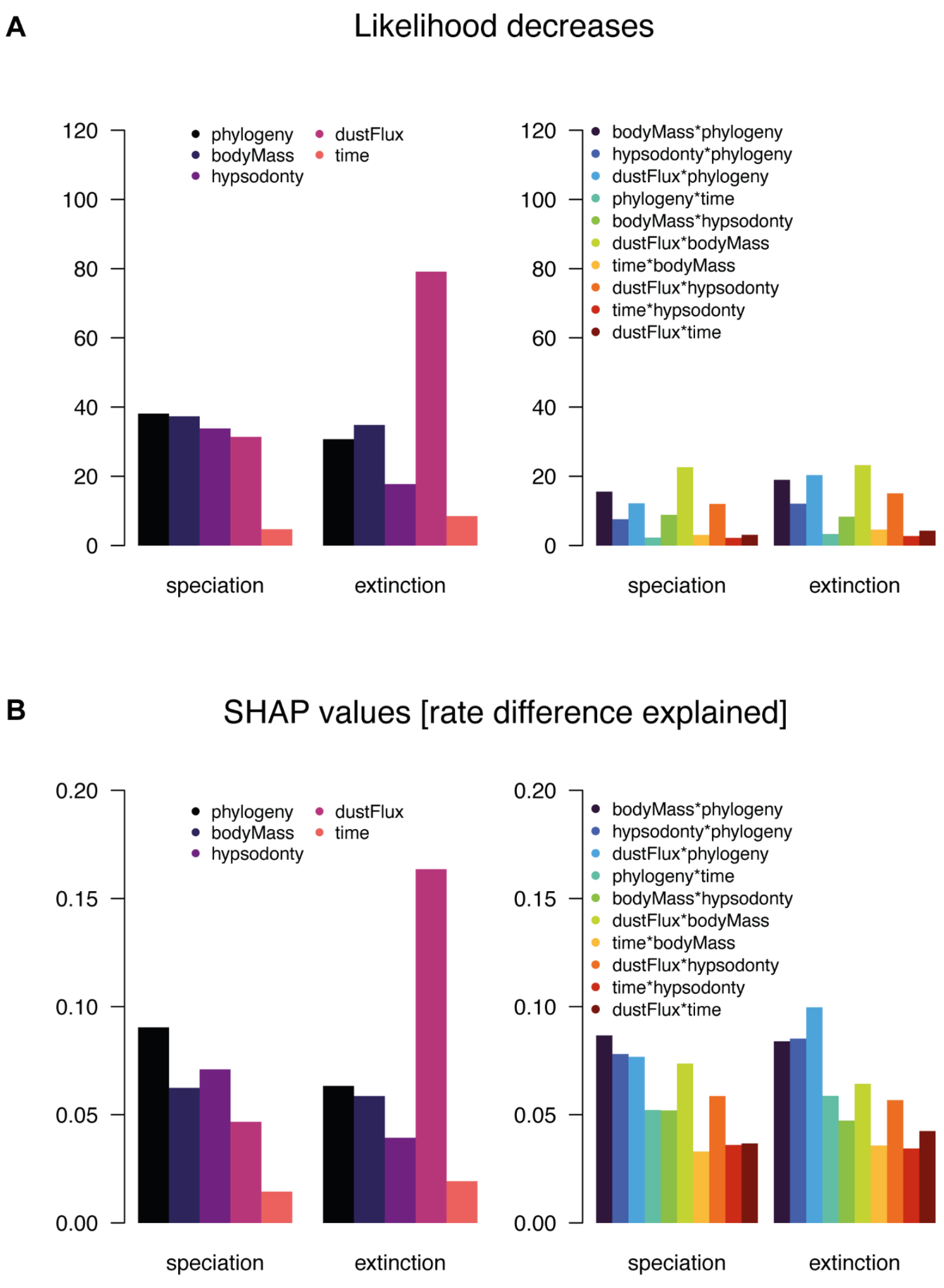

Supplementary Figure 4. Predictors importance of our model with δ^{13}C in long chain *n*-alkanes (C_{35}) as environmental proxy. Importance is measured as the decrease in the model fit ΔLik (after shuffling the values of the focus predictor; **A**) and the contribution to the rate value by each predictor (as SHAP values, **B**).

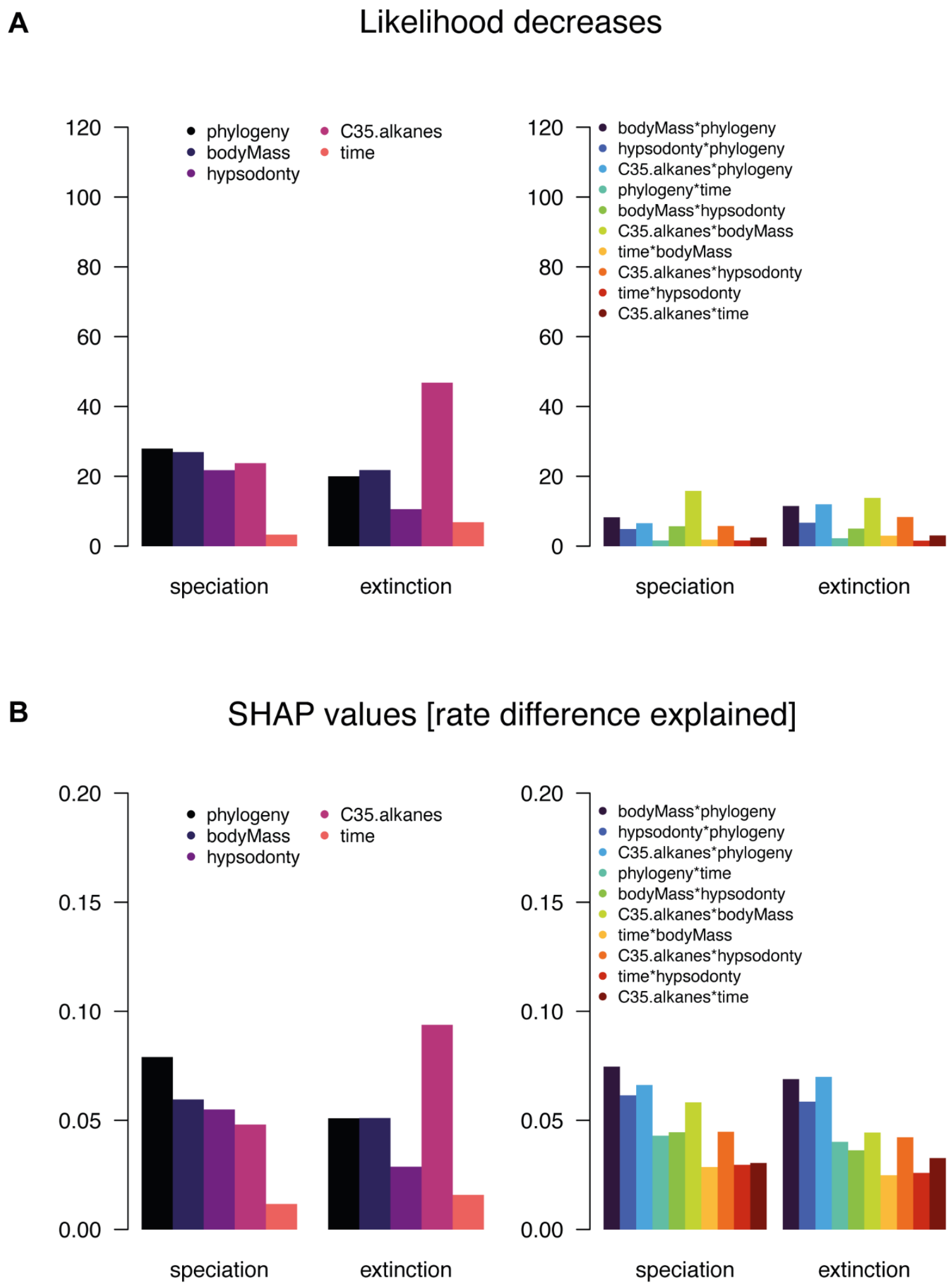

Supplementary Figure 5. Predictors importance of our model with both dust flux and $\delta^{13}C$ in long chain *n*-alkanes (C_{35}) as environmental proxies. Importance is measured as the decrease in the model fit ΔLik (after shuffling the values of the focus predictor; **A**) and the contribution to the rate value by each predictor (as SHAP values; **B**). In each case, only the 10 interactions with the larger values are shown for each rate (in this model there are 15 possible pairs of interactions).

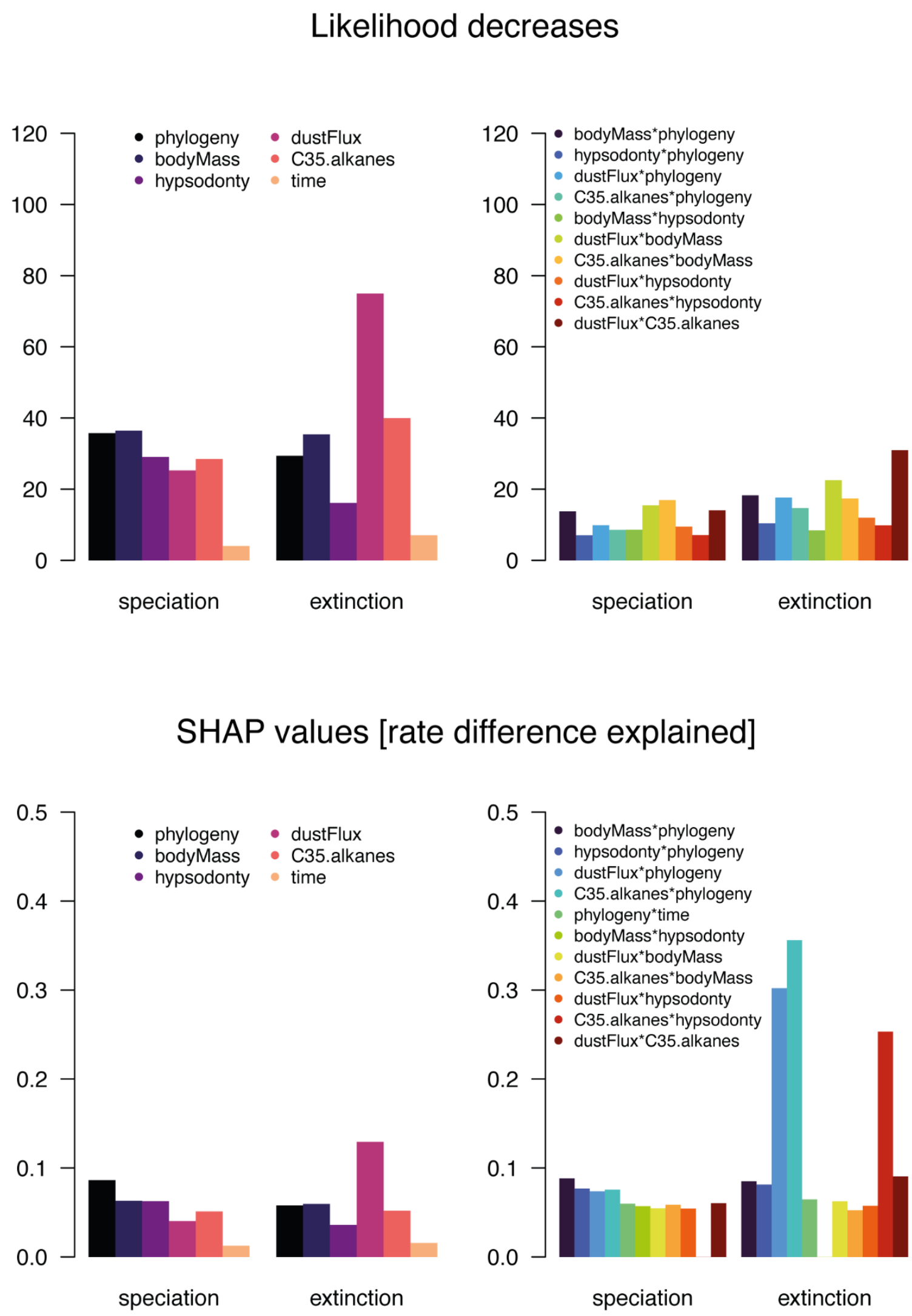

Supplementary Figure 6. Effect δ^{13}C in long chain *n*-alkanes (C_{35}) and traits on diversification. This is a version of Figure 1 but using δ^{13}C in long chain *n*-alkanes (C_{35}) as environmental proxy. Speciation and extinction rates through time in African ungulates (**A**), with δ^{13}C trends at the top [50]. Effect of C_4 grasslands expansion on diversification (**B**). Isolated effect of the interaction of C_4 grasslands expansion and body mass (**C** and **D**), and hypsodonty (**E** and **F**) on speciation (blueish colors) and extinction (reddish colors). Shaded areas represent the 95% highest posterior density (HPD) from 10,000 posterior samples.

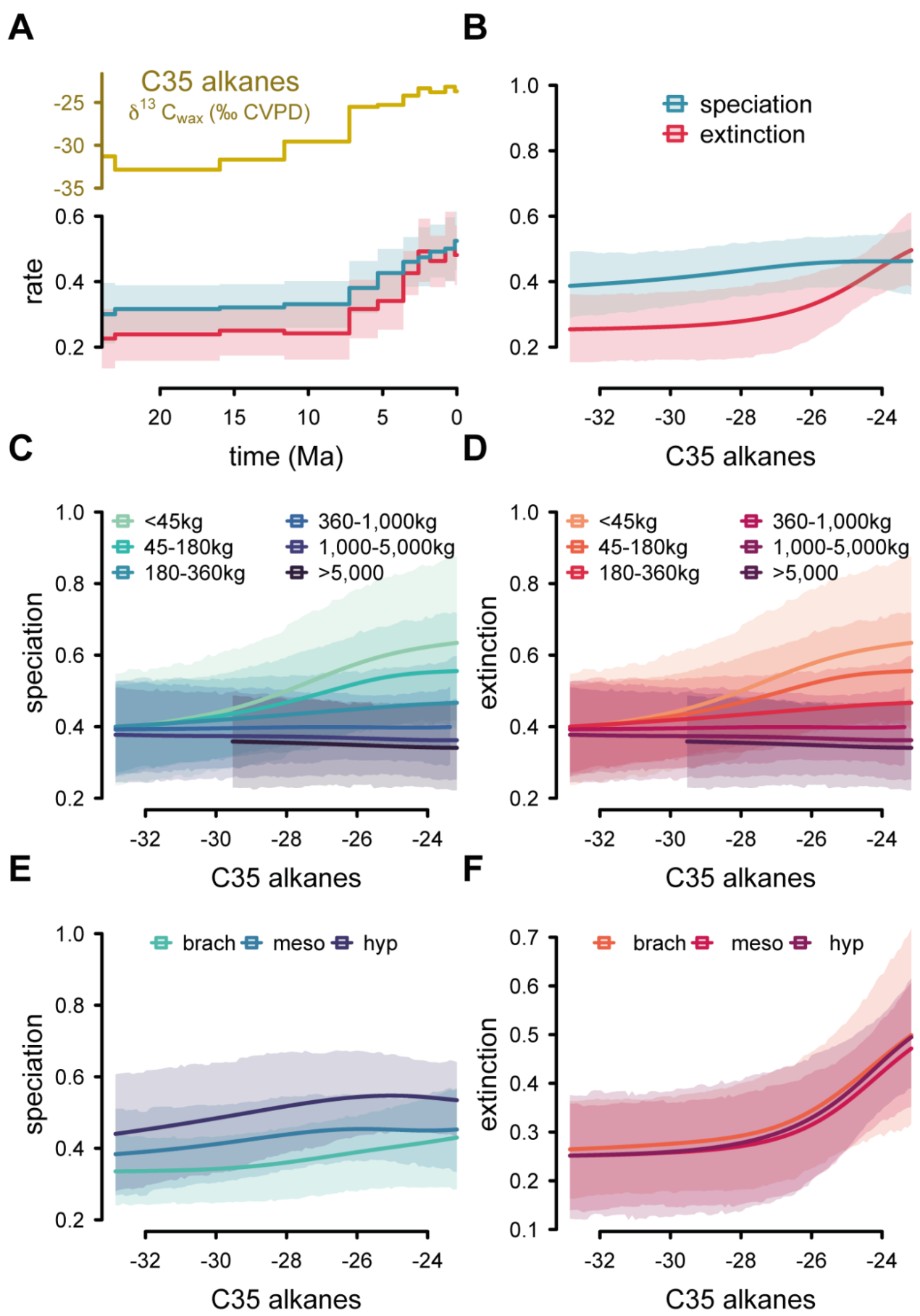

Supplementary Figure 7. Effect of body size and hypsodonty on speciation and extinction rates.

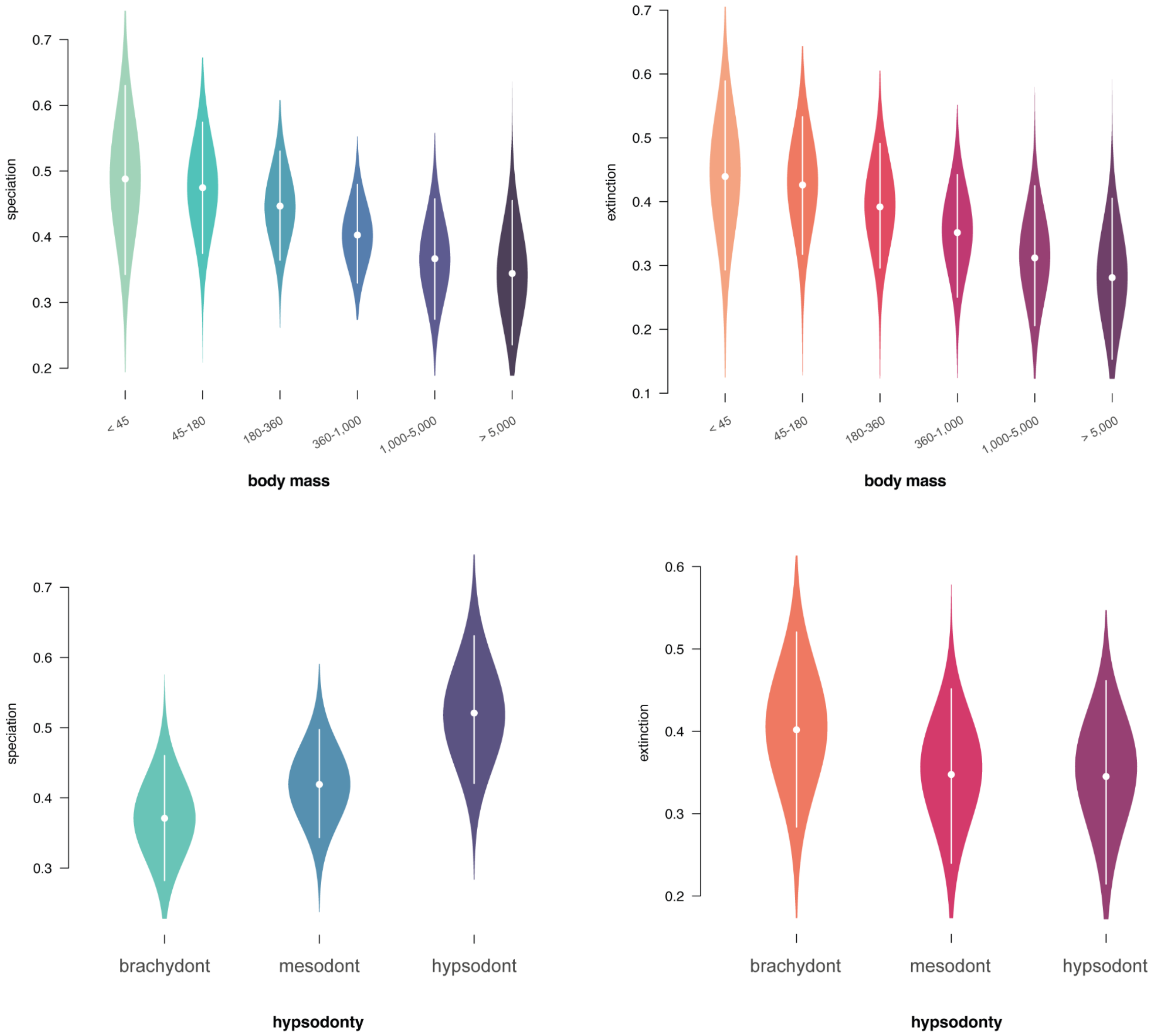

Supplementary Figure 8. Diversification without the effect of aridification. Predicted rates through time across families (same symbols as in Fig. 2) and the ecomorphospace over the last 7.2 Myrs, before and after the Pliocene-Pleistocene boundary (2.58 Ma) where the effect of aridification is marginalized but all other predictors retained their effect. In other words, this is a version of figure 4 in the main text where the effect of aridification is excluded. Ranges of values in colored scales are identical to those in Fig. 3 for an easier comparison. Halving and doubling times are shown over diversification rates for reference. **A**, speciation rates; B, extinction rates; **C**, net diversification rates. Note that net diversification in the last 130 kyrs (last bin, rightmost column) remains positive across lineages and ecomorphologies.

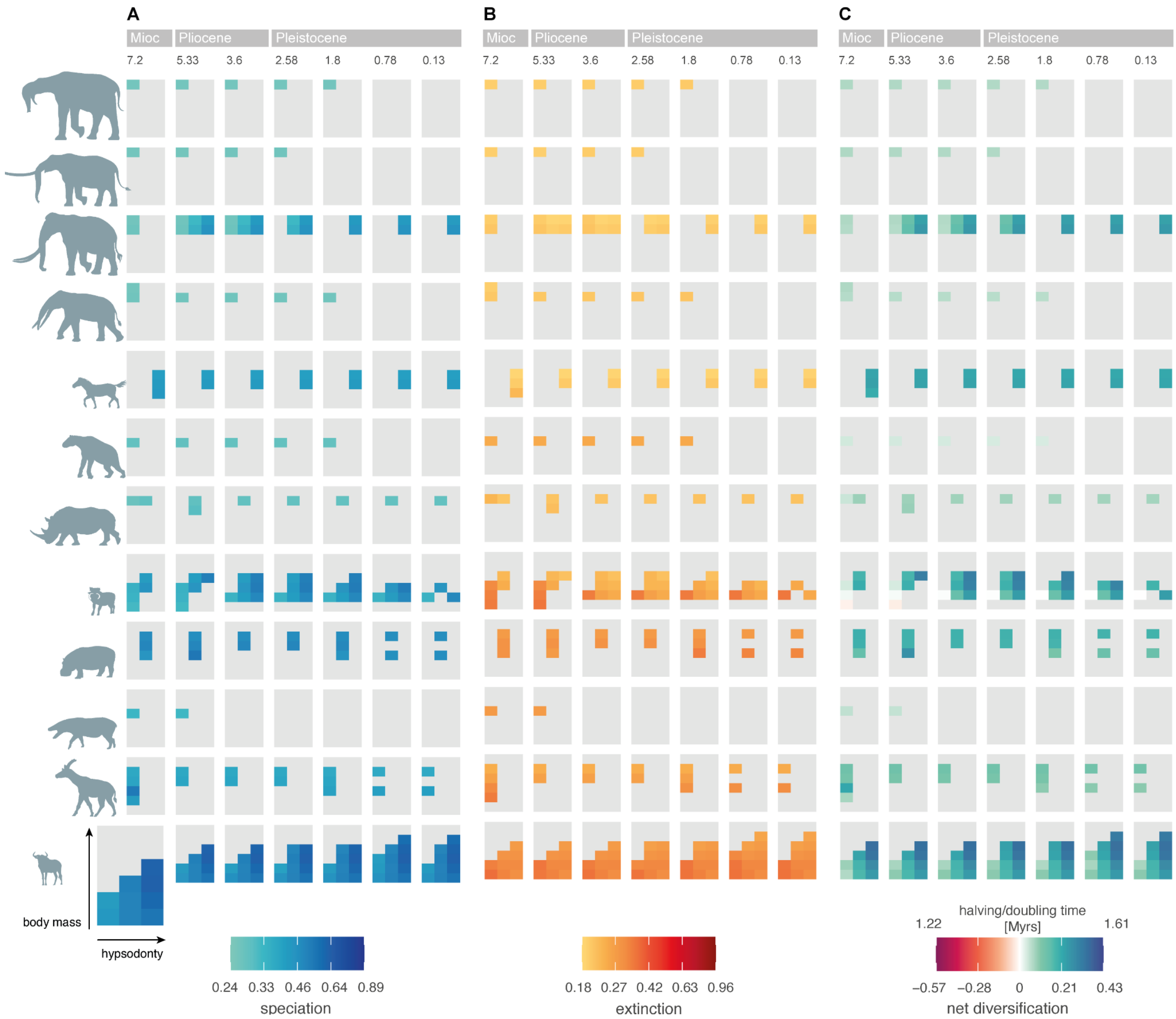

Supplementary Figure 9. Speciation rates over time, lineages (same symbols as in Fig. 2), and ecomorphological gradients in the last 23 Myrs. Rate through time estimates incorporate the effect of all predictors.

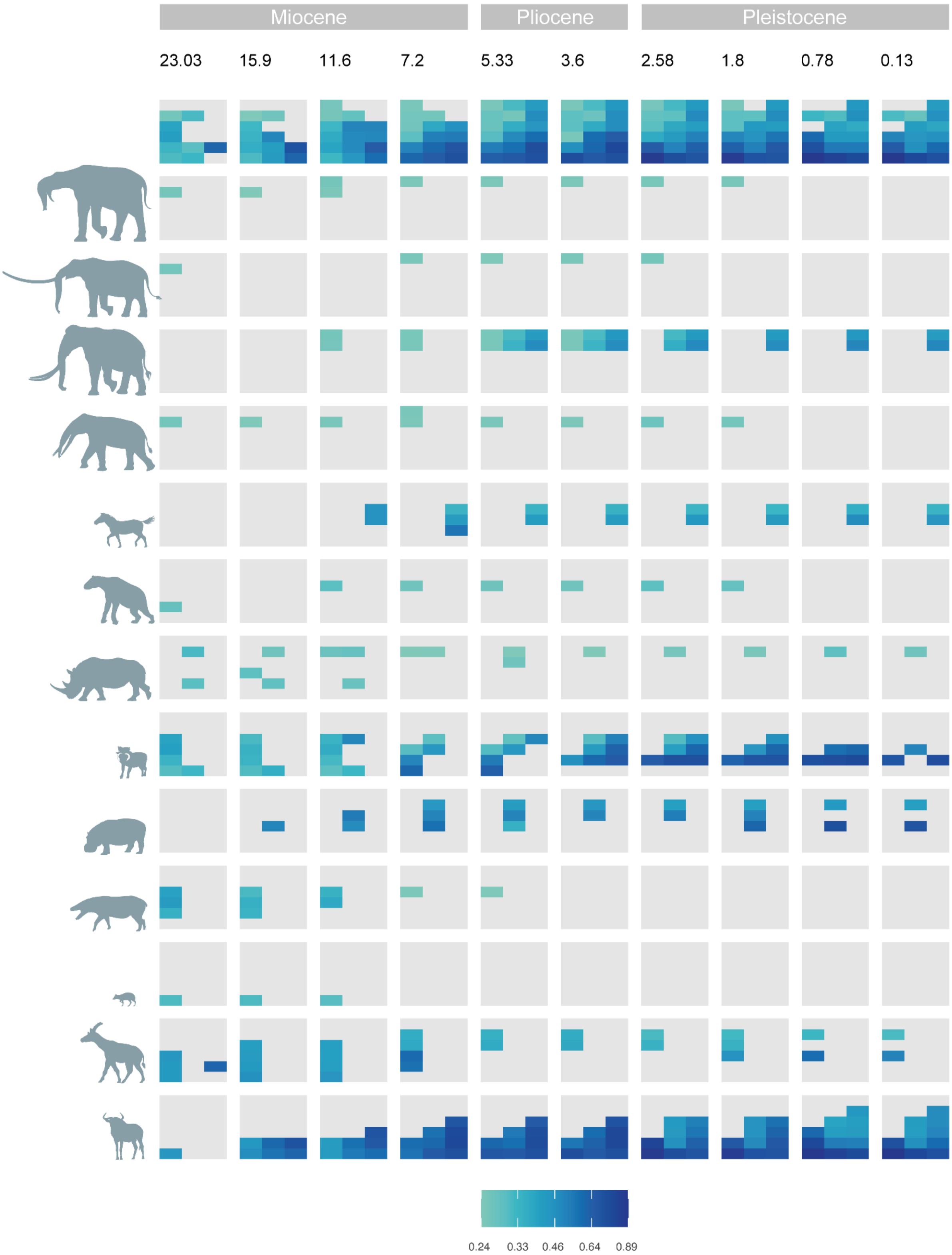

Supplementary Figure 10. Extinction rates over time, lineages (same symbols as in Fig. 2), and ecomorphological gradients in the last 23 Myrs. Rate through time estimates incorporate the effect of all predictors.

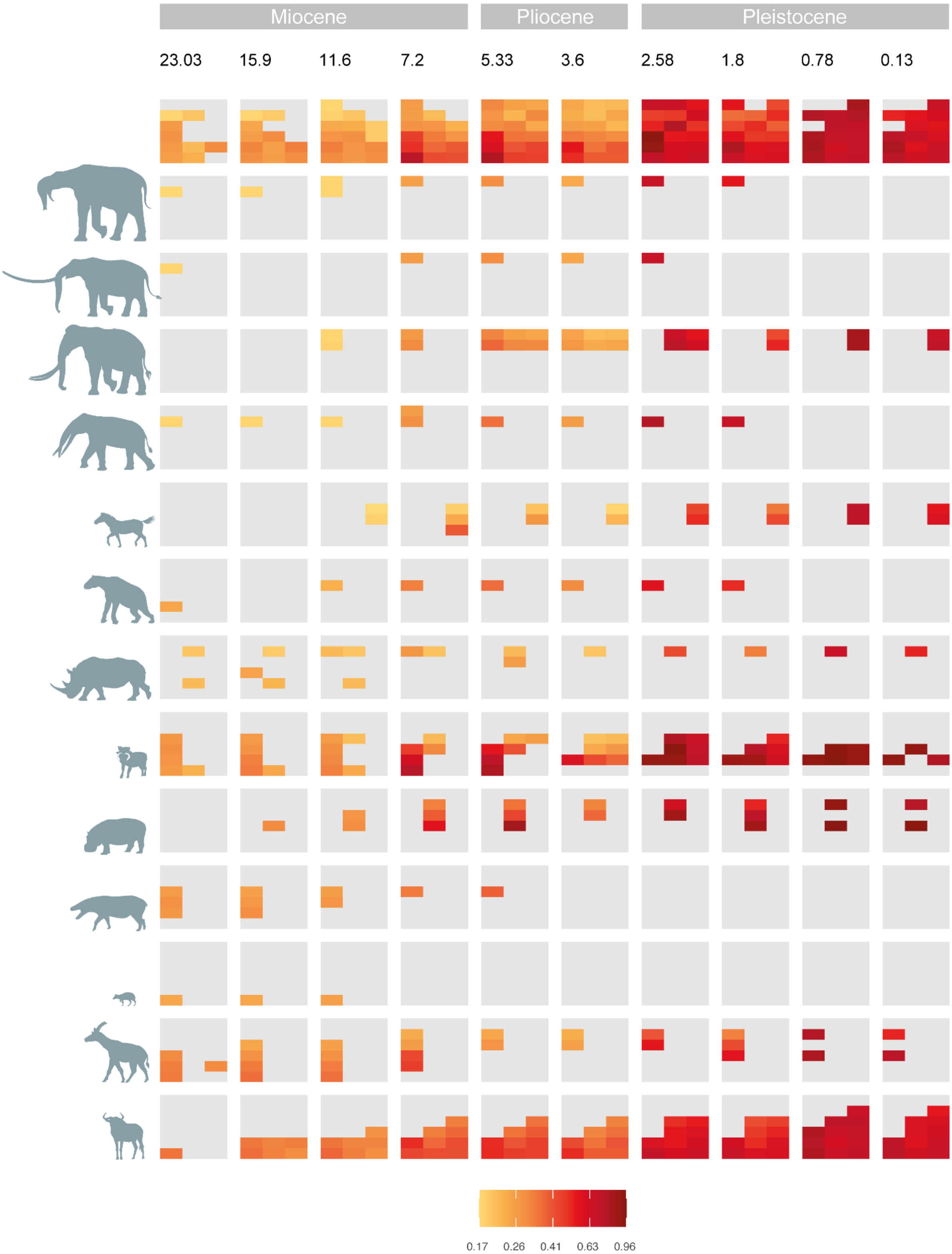

Supplementary Figure 11. Net diversification rates over time, lineages (same symbols as in Fig. 2), and ecomorphological gradients in the last 23 Myrs. Rate through time estimates incorporate the effect of all predictors. Note the different scale in positive and negative values of net diversification.

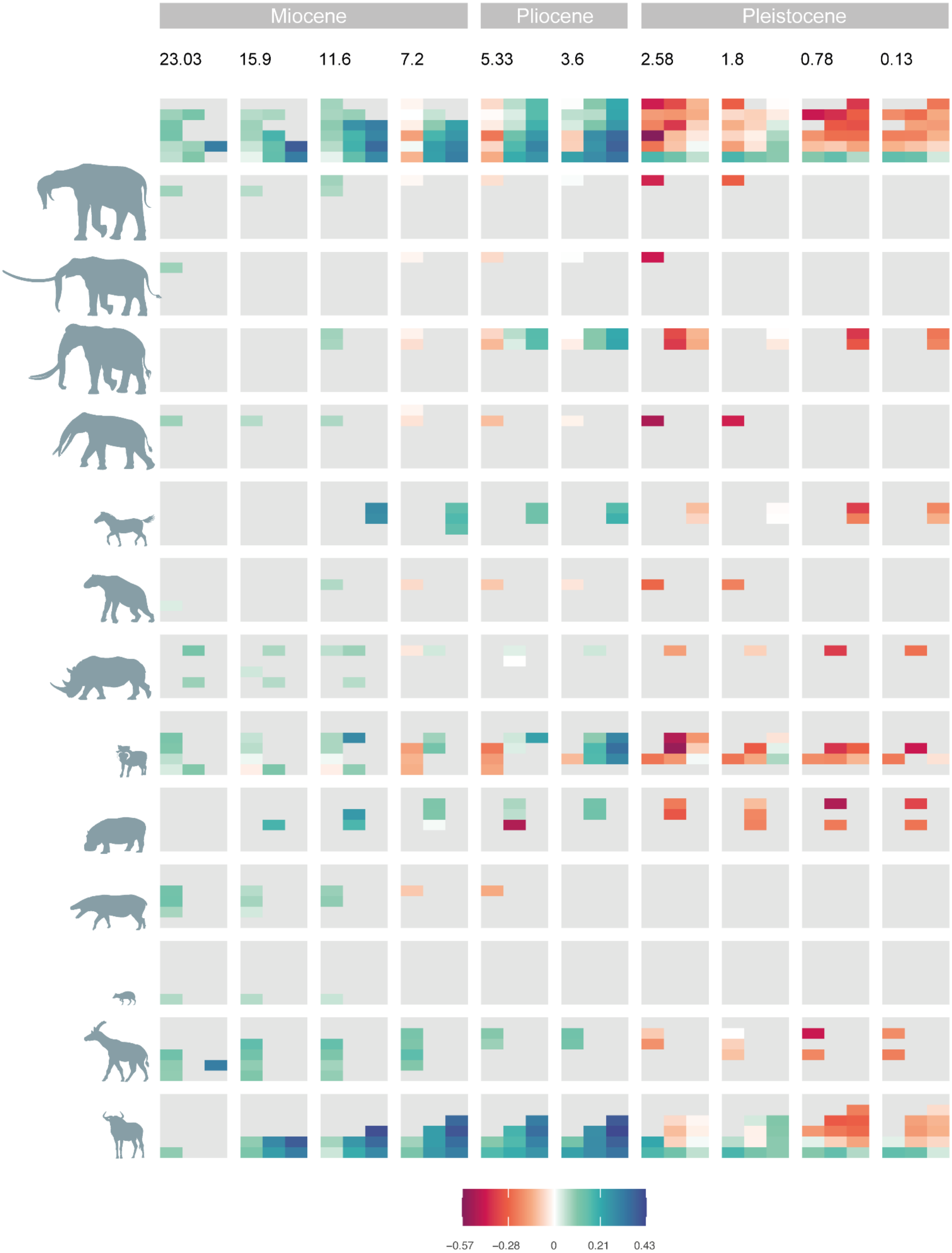

Supplementary Figure 12. Rates through time over the last 10 Myrs estimated for each family except Tragulidae. Note that many families of large-bodied species show a noticeable decrease of speciation starting in the Pliocene, 5.33 Ma. Extinction (red), speciation (blue).

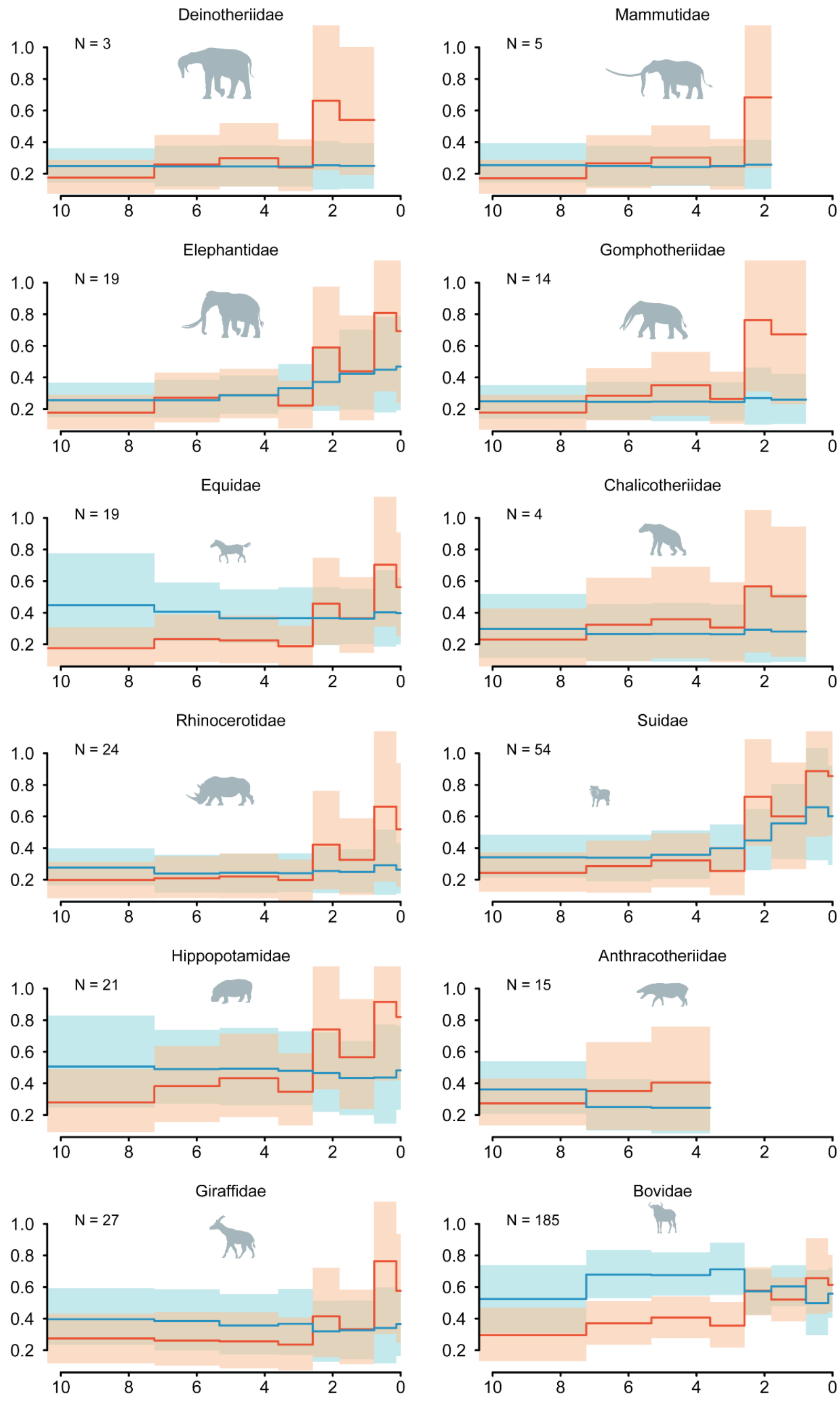

Supplementary Figure 13. Proportion of first appearance records (FADs) of African ungulate species (a) and genera (c) with earlier occurrences outside Africa. A taxon was considered to have a Eurasian origin when its Eurasian FAD predated its African FAD by at least 0.2 Myr, based on the upper age range of individual occurrences. The corresponding family for each Eurasian-origin species and genus is also shown in plates b and d.

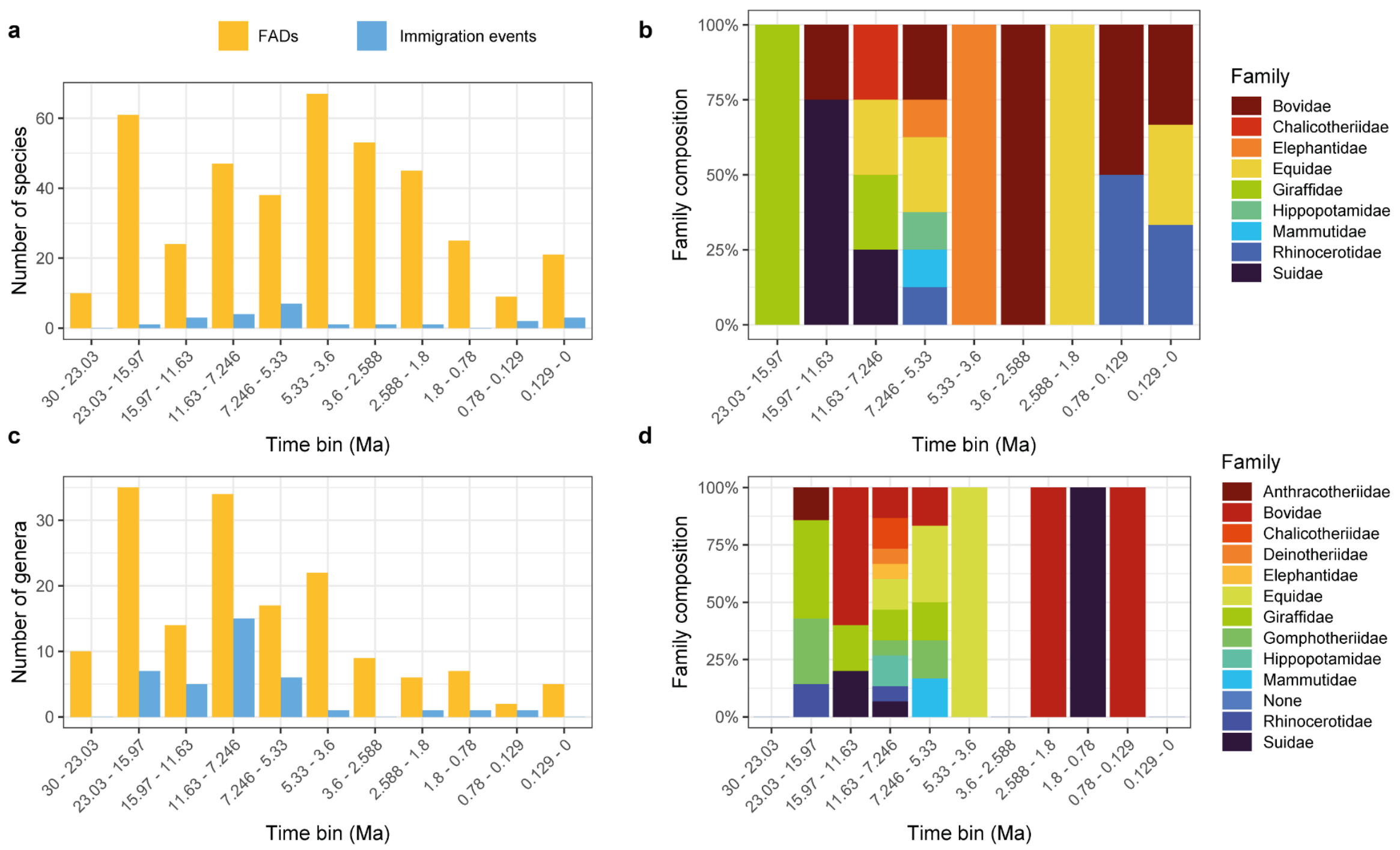

Supplementary Figure 14. Geographical distribution of African localities in each temporal bin used in our analyses.

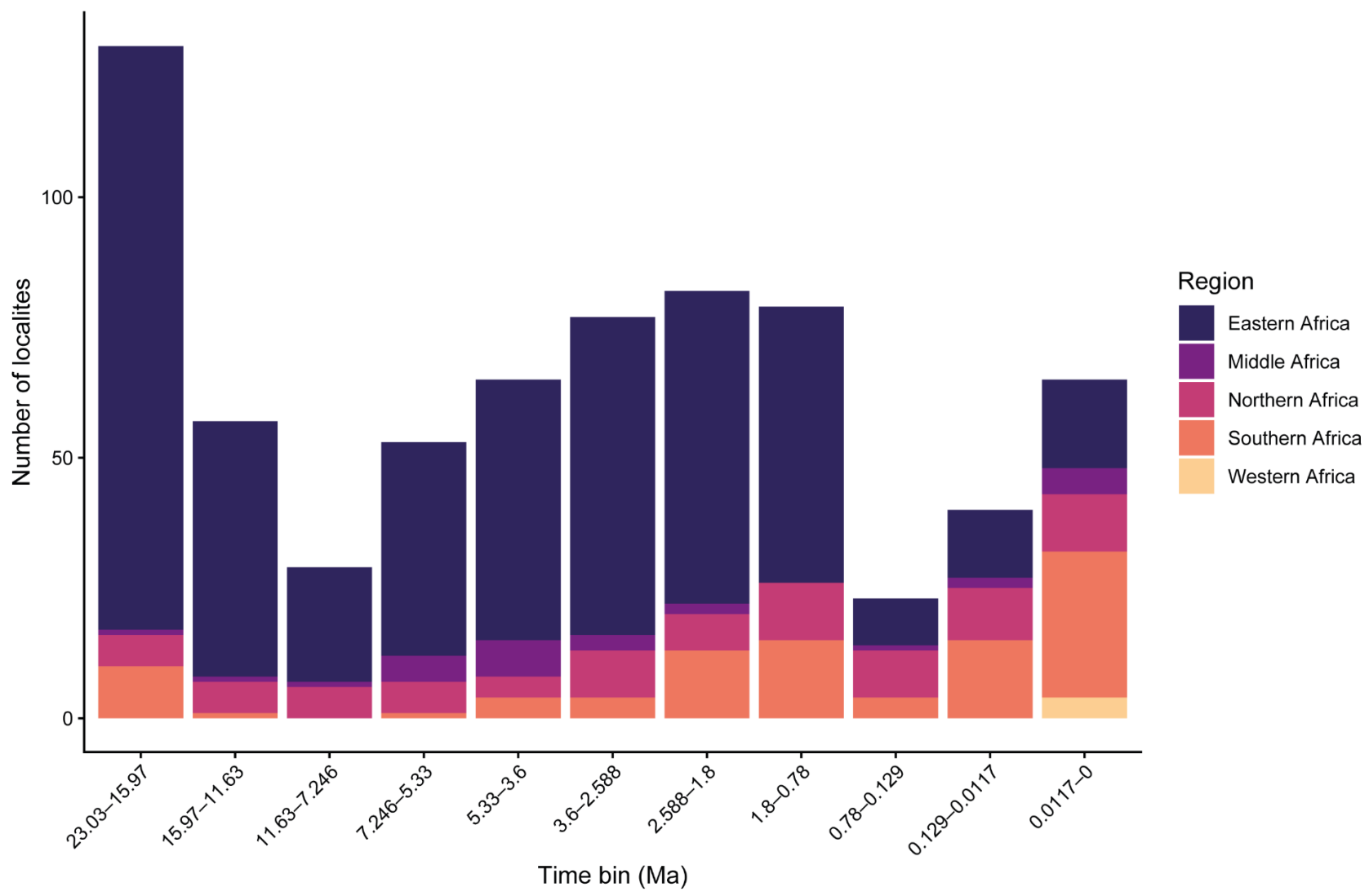

Supplement: Supplementary file 1 [file 41467_2026_76105_MOESM1_ESM.pdf]
