# Supplementary material for: Lower speciation, not higher extinction, drove African megaherbivore diversity collapse
Source: Nat Commun. 2026 Jul 31;17:9373. doi: 10.1038/s41467-026-76105-2 (PMC13534676; doi:10.1038/s41467-026-76105-2)
Supplement: Supplementary file 2 [file 41467_2026_76105_MOESM2_ESM.pdf]

nature portfolio

Peer Review File

Lower speciation, not higher extinction, drove African megaherbivore diversity collapse

Corresponding Author: Dr Juan Cantalapiedra

This file contains all reviewer reports in order by version, followed by all author rebuttals in order by version.

Version 0:

Reviewer comments:

Reviewer #1

(Remarks to the Author)
For decades, paleoecologists have explored how climatic and ecological change shaped Africa's late Cenozoic faunal communities. Recent work highlights that a defining feature of the reorganization of African faunas is the loss of megaherbivore diversity and abundance over the last 4-5 Myr, a trend that parallels the later extinction (i.e., over the last 100 kyr) of megaherbivores across the globe. As noted by the authors, the loss of these ecosystem engineers represents "one of the most profound ecological disruptions in terrestrial ecosystems over the last 30 million years." What drove the demise of Africa's megaherbivores? Some have linked these losses to anthropogenic impacts, but others have demonstrated that these losses begin long before hominins were capable of impacting large-bodied herbivores, instead linking the demise of megaherbivores to bottom-up processes (e.g., C4 expansion or declining primary productivity.)

This study represents a timely and novel contribution to this problem: the role of speciation and extinction in shaping faunal composition over evolutionary time-scales is unknown. This is critical because we do not know if the loss of megaherbivores was a consequence of increased extinction rates (perhaps the implicit assumption in most studies) or instead decreased speciation rates. Here, through a sophisticated analysis of fossil data spanning the last 24 million years, the authors show that increasing aridity (and associated decline in productivity) contributed to a greater decrease in speciation rates in large-bodied lineages—implying that the loss of megaherbivores is related to depressed speciation rather than elevated extinction. They also show that aridity may be a key driver of macroevolutionary processes. I think this is an important study and worth of publication, pending further revision.

1. The spatial scale of the fossil data differ from that of the climatic (dust flux) and environmental (d13C) records used to explore extinction and speciation rates through time. The fossil data are derived from across Africa, whereas the dust flux record derives from a marine core off NW Africa (Polissar et al 2019) and the d13C record derives from the Somali Basin, Gulf of Aden, and Red Sea (Uno et al 2016). This is an example of "scale-jumping" (Behrensmeyer et al 2007), and it assumes that regional-scale climatic/environmental records are representative of Africa, and its fossil record, as a whole. This assumption may be problematic. For example, if the pattern of C4 expansion differs across Africa, then the dataset used here may not be the best predictor of continental-scale macroevolutionary dynamics. This is likely the case. Marine d13C records from SW Africa suggest C4 plant expansion beginning ~8 Ma (Dupont et al 2013), somewhat later than marine core records from elsewhere (Polissar et al 2019). Similarly, terrestrial records from the East African Rift System (EARS), the source of much of the fossil data considered here, suggest a major phase of C4 expansion ~4-5 Ma (Levin 2015), a pattern that is not matched in the marine core records, likely because larger-scale processes captured in the marine records are overprinted by more localized processes in the EARS. If the dust flux record is a better representation of continental-scale climate (and I'm not saying it is…), this could be why aridity emerges as a better predictor of macroevolutionary processes. What to do about this? I see two possible ways forward. Perhaps the ideal approach would be to synthesize all relevant climatic and environmental marine core records to generate a continental-scale composite. This would align your scales by providing continental-scale environmental records that match your continental-scale fossil records. Cohen et al. (2022) provide an example of how this could be done. Alternatively, if that were not feasible for whatever reason, the authors could proceed with the analysis as it is, but they would have to address the assumptions (i.e., that the environmental records are representative of continental-scale climate and environment) and acknowledge potential limitations and implications for interpretation.

2. I would like to see further discussion and emphasis on the dramatic increase in extinction rates over the past 5 Ma (e.g., in

the section entitled “The restructuring of African ungulate faunas”). Looking at the key results figures (e.g., Fig. 1A, Fig. 2, ED Fig 12), the first thing that jumps out is the sharp rise in extinction rates during the Pliocene and Pleistocene for most taxa. The outcome is a net loss of continental-scale diversity over time. This is a really interesting and important result, and one could’ve written an entire paper about that alone. Though it is not the central question, it’s probably worth more attention that it gets in the current manuscript.

3. Throughout the text, the long-term aridification trend is linked to changes in productivity: “long-term aridification trends and the associated changes in productivity”, “likely due to the strong linkage between aridity and primary productivity”, and “prolonged aridification trend and associated declining productivity.” I take no issue with the claim that aridity and productivity are closely linked, but things are a bit complicated from the perspective of large-bodied herbivores. In Africa today, the highest biomass and diversity of ungulates is observed in areas with intermediate rainfall (~600-800 mm/yr; see Fig. 5 and Fig. 6 of Hempson et al 2015). Ungulate diversity and biomass decline at both higher (>800 mm/yr) and lower (<800 mm/yr) rainfall (Hempson et al 2015). This hump-shaped relationship is thought to be a consequence of the interplay between plant biomass, which increases with rainfall, and plant forage quality, which decreases with rainfall (Olff et al 2002). This means that aridification could foster conditions that are beneficial to herbivores (e.g., a decline from 1200 to 800 mm/yr rainfall) or detrimental to herbivores (e.g., a decline from 800 to 400 mm/yr rainfall). What does this complexity mean for the observed relationship between aridity and speciation/extinction rates through time? I’d like to see the authors contend with this in a revised manuscript.

Minor edits/question/comments
Page 2 Line 9: Not until I read the paper did I understand what is meant by “durable dentitions.” Perhaps reword.
ED Fig. 7: Brachydont is spelled incorrectly
Page 4 Line 13-14: “its effect on extinction was delayed and milder in larger size categories” The confidence intervals in Fig 1D are quite broad…is this real?
Page 5 Line 31: “…yielding a better model than one based on C4 grasses.” I do wonder if this may be partially a consequence of spatial variability in the pattern and timing of C4 expansion across Africa (noted above).
Page 5 Line 37: add comma after “Without the influence of aridification[comma]”
Page 6 Line 6-7: “aridification increased the proliferation of hypsodont forms” – can you be clearer about what you mean by proliferation?
Page 6 Line 14: what defines “extreme aridification”?
Page 6 Line 18: “…with widespread effects on herbivore species” Such as?
Conclusions: “Our results reveal that this decline was primarily driven by a prolonged aridification trend and associated declining productivity, rather than solely by Late Pleistocene extinctions…” Huh? Wording here is off. How can a long-term loss of megaherbivores over millions of years be driven by Late Pleistocene extinctions?
ED Fig 3-5: Maybe it's just me, but I have a real hard time differentiating the blue-greens here. Perhaps an alternate color scheme would be helpful? And the font of some of the labels is tiny.

Behrensmeyer AK, Bobe R, Alemseged Z. 2007. Approaches to the analysis of faunal change during the East African Pliocene. In Hominin environments in the East African Pliocene, ed. R Bobe, Z Alemseged, AK Behrensmeyer, pp. 1-24: Springer
Cohen A, Du A, Rowan J, Yost C, Billingsley A, et al. 2022. Plio-Pleistocene environmental variability in Africa and its implications for mammalian evolution. Proceedings of the National Academy of Sciences of the United States of America 119
Dupont LM, Rommerskirchen F, Mollenhauer G, Schefuß E. 2013. Miocene to Pliocene changes in South African hydrology in relation to the expansion of C4 plants. Earth and Planetary Science Letters 375: 408-17
Hempson GP, Archibald S, Bond WJ. 2015. A continent-wide assessment of the form and intensity of large mammal herbivory in Africa. Science 350: 1056-61
Levin NE. 2015. Environment and climate of early human evolution. Annual Reviews of Earth and Planetary Sciences 43: 405-29
Olff H, Ritchie ME, Prins HHT. 2002. Global environmental controls of diversity in large herbivores. Nature 415: 901-5
Polissar PJ, Rose C, Uno KT, Phelps SR, deMenocal P. 2019. Synchronous rise of African C4 ecosystems 10 million years ago in the absence of aridification. Nature Geoscience 12: 657-60
Uno KT, Polissar PJ, Jackson KE, deMenocal PB. 2016. Neogene biomarker record of vegetation change in eastern Africa. 113: 6355-63

Reviewer #2

(Remarks to the Author)
[This review was jointly written by two co-reviewers following the guiding questions for reviewers.]

- What are the noteworthy results?

Using a sophisticated modeling technique to analyze the diversity dynamics of African fossil ungulates over the last 24 million years, the authors of this study found that large body size did not, in general, increase extinction risks of species, but decreased speciation rates, especially as aridification progressed during the Plio–Pleistocene. As a result, the largest-bodied members of the African megafauna had been in decline millions of years before hominins started to exert severe hunting pressure on them.

The reported major impacts of aridity on mammal dynamics are noteworthy because they seem to paint a divergent picture

from some of the recent research on the topic [1–4]. These previous studies found no connection between herbivore paleodiet and aridity changes through the Pliocene-Pleistocene [1], nor changing vegetation with aridity over the past ten million years [4].

- Will the work be of significance to the field and related fields? How does it compare to the established literature? If the work is not original, please provide relevant references.

This work is significant in its current form as it brings much needed attention back to the speciation side of the history of African mammals—a subject that was central to Vrba's evolutionary hypothesis [5, 6]. Its impact will be felt by those who study ungulates, east African fauna, and hominin evolution in the context of changing environments around its origin.

The significance of this study will be further enhanced if it manages to present a mechanistic synthesis of differing ways in which aridity impacts body size, diet, and diversification. To be fair, the authors do offer some explanatory hypotheses for the observed rate patterns. However, without explicit consideration of alternative hypotheses, those potential explanations give an impression of having been formulated after the fact, even though they are presented as predictions (P. 3, Lines 24–34), and are rather confusing in some places. For example, as aridification progressed, hypsodont taxa are said to "have encountered more spatially homogeneous resource distributions" (P. 7, Line 5) resulting in their lower extinction rates compared to brachydont taxa, and at the same time, experienced higher speciation rates "under local adaptive pressures"-- But wouldn't homogeneous resource distribution make local adaptive pressures homogenous as well, suppressing speciation? Elaborating on these potential explanations in connection with different modes of speciation (allopatric/peripatric/parapatric) may help make the authors' arguments more convincing. Overall, we feel that an expanded and clearer discussion of why diversification patterns differed among ecomorphs would make this work more impactful. In addition, a conceptual diagram or a table of proposed mechanisms would help the reader better understand the implications of the results.

Concerning previous research connecting increasing aridity and biotic dynamics of Eastern Africa, the lack of change in enamel isotope values with increasing aridity [1], suggests that mammals may still have been locating their preferred foods (possibly in heterogeneous environments) even in a continental shift to increased aridity. Alternatively, the proxy of aridity (dust flux) may not be a faithful indicator of the vegetation dynamics on the continent. This latter point was at least in part the conclusion of the lack of association in increasing C4 grasslands with increasing aridity [4]. Both of these previous studies highlighted decoupling of aridity from biotic dynamics, in contrast to the findings of the present paper. We encourage the authors to reconcile their findings with those of the previous studies, elaborating on why aridification drove diversity dynamics without major shifts in vegetation or ungulate diet.

- Does the work support the conclusions and claims, or is additional evidence needed?

There is one specific conclusion that we think requires additional scrutiny, which is that dietary flexibility suppressed extinction rates (P. 3, Lines 36-37). Extended Figure 7 suggests that hypsodonty and mesodonty are nearly identical in distribution of extinction rates, while only brachydonty appears greater. This suggests that there may be a threshold of crown height that facilitates dietary diversity, but the tallest crown heights do not grant ungulate species additional benefit of increased survival. Likewise, hypsodont and mesodont taxa respond nearly identically to increasing aridity (Fig. 1f). On the other hand, there appears to be a monotonic trend of increasing speciation rate with greater crown height (Extended Figure 7). Could the authors explain why there might be a threshold crown height for extinction but not speciation?

- Are there any flaws in the data analysis, interpretation and conclusions? - Do these prohibit publication or require revision?

Although we appreciate the strength of the 'big data' analyses by the authors, we are concerned by the absence of geographic context in this study, which treats the whole continent of Africa as a single system. We think there are at least two problems that need to be addressed in relation to geography.

First, it is unclear to what extent the "speciation" rates estimated in this study pertain to true speciation events in Africa versus immigrations from Eurasia. The authors appear to presume that environmental forces were the key process driving the in-situ speciation, extinction, and diversification of taxa, but there was considerable faunal interchange between Africa and Eurasia during the Neogene [7]. We suggest that the authors mark immigration pulses on the diversity plots. These pulses may be at the African Land Mammal Age (ALMA) boundaries, given that's a primary way in which LMAs are defined. If so, it's unlikely that it will affect interpretation since the authors ran a version of their analysis already using these boundaries, and found it was a worse fit than when using global geological divisions. However, the immigration factor should still be acknowledged, and how it affects interpretation, if at all, should be discussed.

Second, the highly uneven geographic distribution of major Neogene fossil sites in Africa [3, 8] could, in our opinion, distort some of the diversity patterns reported in this study. For example, had the fossil sampling been much better in west and central Africa--regions that could have provided important refugia for forest-adapted taxa (see e.g., [9])--wouldn't the extinction rates for brachydont taxa be lower? We understand that sampling heterogeneity through time was explicitly modeled in BDNN/PyRate, but whether that is sufficient to overcome sampling heterogeneity across environmentally uneven space is unclear to us. Related to this issue, we think it will be helpful to the reader to see a graphical representation of how much data came from which parts of Africa and which time periods.

Overall, we do not think these omissions prohibit publication of this study, but we do think that a revised version of this paper that carefully addresses them will be a more valuable contribution to science.

- Is the methodology sound? Does the work meet the expected standards in your field?

We think the core method is very sound and exceeds the expected standards in the field of paleobiology.

- Is there enough detail provided in the methods for the work to be reproduced?

The methods are thoroughly described, and we greatly appreciate the inclusion of the commands and outputs of the BDNN model, almost to a tutorial degree, which fills in details that do not currently exist on the developers website or in other documentations. Nevertheless, we were unable to check the reproducibility of the study using the provided R script because of this function, which appeared custom-made:
separa_caracteres(
We encourage the authors to (1) provide the function so that others can reproduce the results and even cite the code for inspiration in their own analyses and (2) check again that the rest of the script is capable of being run by someone downloading it as a supplemental material.

References

1. S. A. Blumenthal, N. E. Levin, F. H. Brown, J. P. Brugal, K. L. Chritz, J. M. Harris, G. E. Jehle, T. E. Cerling, J. O'Connell, Aridity and hominin environments. Proc Natl Acad Sci U S A 114, 7331–7336 (2017).

2. N. E. Levin, Environment and climate of early human evolution. Annu Rev Earth Planet Sci 43, 405–429 (2015).

3. J. T. Faith, J. Rowan, A. Du, Late Cenozoic Faunal and Ecological Change in Africa. Annu Rev Earth Planet Sci 52, 379–407 (2024).

4. P. J. Polissar, C. Rose, K. T. Uno, S. R. Phelps, P. deMenocal, Synchronous rise of African C4 ecosystems 10 million years ago in the absence of aridification. Nat Geosci 12, 657–660 (2019).

5. Vrba, Elisabeth S. "Ecology in relation to speciation rates: some case histories of Miocene-Recent mammal clades." Evolutionary Ecology 1, no. 4 (1987): 283-300.

6. Vrba, Elisabeth S. "Mammals as a key to evolutionary theory." Journal of mammalogy 73, no. 1 (1992): 1-28.

7. J. A. Van Couvering, E. Delson, African Land Mammal Ages. J Vertebr Paleontol 40 (2020).

8. Werdelin, Lars. "Chronology of Neogene mammal localities." Cenozoic mammals of Africa (2010): 27-44.

9. Ntie, Stephan, Anne R. Davis, Katrin Hils, Patrick Mickala, Henri A. Thomassen, Katy Morgan, Hadrien Vanthomme, Mary K. Gonder, and Nicola M. Anthony. "Evaluating the role of Pleistocene refugia, rivers and environmental variation in the diversification of central African duikers (genera Cephalophus and Philantomba)." BMC Evolutionary Biology 17, no. 1 (2017): 212.

Reviewer #3

(Remarks to the Author)
I co-reviewed this manuscript with one of the reviewers who provided the listed reports. This is part of the Nature Communications initiative to facilitate training in peer review and to provide appropriate recognition for Early Career Researchers who co-review manuscripts.

Version 1:

Reviewer comments:

Reviewer #1

(Remarks to the Author)
I appreciate the opportunity to examine the revised version of this manuscript. Overall, I believe that the authors have made a good effort to address the reviewer concerns, resulting in a stronger paper. The revisions are appropriate, and the few rebuttals are quite reasonable. I have mostly minor edits to offer on this draft:

Line 145: this is the first use of the abbreviation HPD. Please define it.

Line 247: this wording is somewhat awkward. Suggest rephrasing. Perhaps "In most iterations (90%), the effect of aridification on taxa below 360 kg differs from larger-bodied taxa." And differs in what way? Can you be more specific?
Line 249: previously you spelled "ton" as "tonne" (on line 62)
Line 261: I'd change "first expansion" to "initial expansion" since "First" is used just a few words earlier.
Line 278: change "aridification proxy" to "aridity proxy"
Line 279: change "dryness" to "moisture availability"
Line 279-283: Here you argue that aridity proxy may outperform the C4 grass proxy because the former may capture other important ecological feedbacks (e.g., veg structure, fire, herbivory). But might the same also be true of the C4 grass proxy? Almost certainly the expansion of C4 grasses is associated with changes in fire regimes, herbivory, etc (and likely also encompasses some aspects of climate too).
Line 351: change "brachiodont" to "brachydont'
Line 410-412: I still don't follow this sentence: "This scenario contrasts with hypotheses attributing megaherbivore decline primarily to Late Pleistocene extinctions, which, as we show, lacked size or dietary selectivity" A long-term decline of megaherbivores cannot be a attributed to something that happened within the last ~100,000 years. Do you mean "…decline to the same mechanisms responsible for Late Pleistocene extinctions…"?

Reviewer #2

(Remarks to the Author)
[This review was jointly written by two co-reviewers.]

We thank the authors for their careful consideration of our comments and suggestions, at times even going beyond the recommendations we outlined. Overall, we find substantial improvement to this manuscript with changes that will increase its impact and accessibility to readers.

At this stage, there are a few minor issues that should be addressed before the paper is published:

1) Line 213: If we understand correctly, Reviewer 1 was concerned about spatial mismatch between the faunal data and the paleoenvironmental proxy data, rather than spatial averaging of the latter; i.e., the potential problem is more about bias than scale. For the sake of transparency, it will be good to state (as Reviewer 1 pointed out) where geographically the proxy data come from and discuss to what extent the authors think the proxy datasets reflect continental trends.

2) Extended Data Fig. 13, and the manuscript text assessing the impact of Eurasian immigrant taxa on African diversification dynamics is an exciting addition, though we were confused as to aspects of interpreting the figure. We feel that neither the figure caption nor the manuscript text provides adequate context as to what the "Immigration" and "FADs" data points represent. The authors' response to our initial comments states "...for each temporal bin, we quantify the fraction of new occurrences in Africa that correspond to species and genera previously sampled in Eurasia." Combined with a text example of time bin 7.2 to 5.33 Ma having three species immigrating from Eurasia, this should correspond to the blue bar "FADs" data. What then is the "Immigration" data? Is it overall first appearances in Africa during that time bin? If so, shouldn't these labels be the other way around?

Please also provide more information on how immigrants from Eurasia to Africa (as opposed to those from Africa to Eurasia) were identified. For example, does "previously sampled in Eurasia" mean occurrence in an earlier time bin? If so, taxon name-matching may, once again, underestimate immigration events when individual time bins have long spans, so that should be acknowledged.

3) We are concerned that the species-level name-matching between Eurasia and Africa may substantially underestimate the true frequency of immigration events. Unless the temporal density of the fossil dataset is very high, species name-matching is unlikely to capture many of the immigrant Eurasian taxa when they first appear in Africa, since there is a sensible expectation that geologically rapid local adaptation of immigrants will result in paleontologists recognizing them as new species, even though they are part of the same lineages that came from Eurasia. Our suggestion is to avoid this issue by focusing more on the genus-level data that the authors have already included, with additional contextual information for the reader in the form of figure caption or manuscript text as described above.

The following are minor editorial notes:
Line 244: "speciation by around 50% rates" -> "speciation rates by around 50%"
Line 399: "buffered against extinction rates" -> "buffered against extinction"

In closing, we look forward to publication of this important study, and hope that future studies in this line of research involve more direct participation of researchers from (or based in) African countries, following models developed by field-based research communities.

Reviewer #3

(Remarks to the Author)
I co-reviewed this manuscript with one of the reviewers who provided the listed reports. This is part of the Nature Communications initiative to facilitate training in peer review and to provide appropriate recognition for Early Career Researchers who co-review manuscripts.

Open Access This Peer Review File is licensed under a Creative Commons Attribution 4.0 International License, which permits use, sharing, adaptation, distribution and reproduction in any medium or format, as long as you give appropriate credit to the original author(s) and the source, provide a link to the Creative Commons license, and indicate if changes were made.
In cases where reviewers are anonymous, credit should be given to 'Anonymous Referee' and the source.
The images or other third party material in this Peer Review File are included in the article's Creative Commons license, unless indicated otherwise in a credit line to the material. If material is not included in the article's Creative Commons license and your intended use is not permitted by statutory regulation or exceeds the permitted use, you will need to obtain permission directly from the copyright holder.
To view a copy of this license, visit https://creativecommons.org/licenses/by/4.0/

RESPONSE TO REVIEWERS' COMMENTS

All authors agree that the reviewers' comments and references have been extremely helpful, and we have carefully addressed all points raised. As part of the revision process, we have re-run all models and analyses. This was prompted by the identification of a minor bug in the BDNN model code, whose correction has led to a clearer understanding of why the model driven by the aridity proxy outperforms the alternative proxy related to plant expansion; this is now explained in greater detail in the revised manuscript. Importantly, despite re-running all analyses with the corrected code, the overall correlations and main conclusions remain unchanged.

We have also ensured that all correlations and rankings of predictor importance are interpreted consistently with the temporal focus of the study: although the fossil record used in the analyses extends back to approximately 30 million years ago, all quantitative interpretations are now explicitly restricted to the last 23 million years, which constitute the focal interval of our study. The updated results indicate that, although increasing aridity is still associated with a predicted decline in speciation rates of the largest-bodied taxa of approximately 20%, this effect is somewhat more moderate than in the previous version. In light of this, we have decided to adopt a more conservative title, while fully retaining and discussing this decline where it was relevant.

Reviewers' comments are shown in black and our responses are in green. Modified sections in the manuscript are also highlighted in green.

Thank you for your time and consideration,

Juan L. Cantalapiedra

Reviewer 1

For decades, paleoecologists have explored how climatic and ecological change shaped Africa's late Cenozoic faunal communities. Recent work highlights that a defining feature of the reorganization of African faunas is the loss of megaherbivore diversity and abundance over the last 4-5 Myr, a trend that parallels the later extinction (i.e., over the last 100 kyr) of megaherbivores across the globe. As noted by the authors, the loss of these ecosystem engineers represents "one of the most profound ecological disruptions in terrestrial ecosystems over the last 30 million years." What drove the demise of Africa's megaherbivores? Some have linked these losses to anthropogenic impacts, but others have demonstrated that these losses begin long before hominins were capable of impacting large-bodied herbivores, instead linking the demise of megaherbivores to bottom-up processes (e.g., C4 expansion or declining primary productivity.)

This study represents a timely and novel contribution to this problem: the role of speciation and extinction in shaping faunal composition over evolutionary time-scales is unknown. This is critical because we do not know if the loss of megaherbivores was a consequence of increased extinction rates (perhaps the implicit assumption in most studies) or instead decreased speciation rates. Here, through a sophisticated analysis of fossil data spanning the last 24 million years, the authors show that increasing aridity (and associated decline in productivity) contributed to a greater decrease in speciation rates in large-bodied lineages—implying that the loss of megaherbivores is related to depressed speciation rather than elevated extinction. They also show that aridity may be a key driver of macroevolutionary processes. I think this is an important study and worth of publication, pending further revision.

We sincerely thank the reviewer for the positive comments and for recognizing our work as a valuable contribution to understanding the impact of environmental changes on African mammals.

Comment 1.1. The spatial scale of the fossil data differ from that of the climatic (dust flux) and environmental (d13C) records used to explore extinction and speciation rates through time. The fossil data are derived from across Africa, whereas the dust flux record derives from a marine core off NW Africa (Polissar et al 2019) and the d13C record derives from the Somali Basin, Gulf of Aden, and Red Sea (Uno et al 2016). This is an example of "scale-jumping" (Behrensmeyer et al 2007), and it assumes that regional-scale climatic/environmental records are representative of Africa, and its fossil record, as a whole. This assumption may be problematic. For example, if the pattern of C4 expansion differs across Africa, then the dataset used here may not be the best predictor of continental-scale macroevolutionary dynamics. This is likely the case. Marine d13C records from SW Africa suggest C4 plant expansion beginning ~8 Ma (Dupont et al 2013), somewhat later than marine core records from elsewhere (Polissar et al 2019). Similarly, terrestrial records from the East African Rift System (EARS), the source of much of the fossil data considered here, suggest a major phase of C4 expansion ~4-5 Ma (Levin 2015), a pattern that is not matched in the marine core records, likely because larger-scale processes captured in the marine

records are overprinted by more localized processes in the EARS. If the dust flux record is a better representation of continental-scale climate (and I'm not saying it is…), this could be why aridity emerges as a better predictor of macroevolutionary processes.

What to do about this? I see two possible ways forward. Perhaps the ideal approach would be to synthesize all relevant climatic and environmental marine core records to generate a continental-scale composite. This would align your scales by providing continental-scale environmental records that match your continental-scale fossil records. Cohen et al. (2022) provide an example of how this could be done. Alternatively, if that were not feasible for whatever reason, the authors could proceed with the analysis as it is, but they would have to address the assumptions (i.e., that the environmental records are representative of continental-scale climate and environment) and acknowledge potential limitations and implications for interpretation.

Response 1.1 We thank the reviewer for bringing this issue to our attention. In the revised manuscript, we have substantially expanded the section discussing the role of environmental predictors to clarify the rationale behind our choice of proxies. While we acknowledge that previous studies have successfully combined multiple environmental proxies for Africa, this approach is only feasible when all proxies span the same temporal range (proxy values are available for all bins). As we now explain in detail in the main text, our goal was to contextualize the decline of African megaherbivores not only during the Plio–Pleistocene, but across the entire Neogene and Quaternary. For this reason, we selected the two environmental proxies that consistently cover this extended time interval.

We also explicitly discuss the issue of spatial averaging, as suggested by the reviewer, and we now acknowledge in the manuscript the main limitations inherent to this approach when using continental-scale proxies. As noted in the revised text, current implementations of models such as the one used here do not allow the simultaneous inclusion of environmental data derived from multiple basins or regions. We agree that the ability to incorporate spatially explicit environmental information within such flexible modeling frameworks would represent a major methodological advance, and we highlight this as an important avenue for future work. We touch on these issues both in the *Environmental change and ungulate diversification* section and at the end of the discussion.

Page 6 Line 11. "These changes are well documented in the African paleoenvironmental record, which integrates diverse sources (e.g. isotopes from paleosols, tooth enamel and leaf wax, pollen, dust deposits, etc) from continental outcrops as well as lacustrine and marine cores[47]. This proxy richness offers insight into both the temporal heterogeneity and the spatial variability of African environments during the Late Neogene and Quaternary. However, most paleoenvironmental datasets typically span the last 10 Myrs, and show consistent sampling during the last 5 Myrs[48]. This limits their integration into broader-scale analyses, such as the one undertaken here, which aims to model 23 million years of ungulate diversity dynamics in Africa. We therefore focus on two major aspects of environmental change that are consistently represented across this extended timeframe: increasing aridification [based on rates of dust

flux[12]], and the expansion of C_4 grasses [based on $\delta^{13}C$ isotopic signal in long chain *n*-alkanes (C_{35})[49]]. Although their interdependence remains incompletely understood, aridity shapes the distribution, spatial variability, and abundance of vegetation types, and both are linked to complex feedbacks involving seasonality, herbivores pressure and fire regimes[50,51]. Both proxies derive from marine cores, and likely reflect aggregated trends at the subcontinental scale. While we interpret them as capturing the dominant environmental transitions across Africa, we acknowledge their spatial averaging and the need for future reconstructions that integrate basin- or region-specific records to resolve intra-continental heterogeneity in a unifying framework, something that current macroevolutionary models do not yet accommodate.

We ran two separate models using each of these proxies (dust flux and $\delta^{13}C$ isotopic signal in long chain n-alkanes) as the primary environmental predictor, along with a combined model incorporating both variables (see Methods). The model incorporating dust flux as proxy accounted for more variation in speciation and extinction rates across species (Extended Data Fig. 3 and 4; see Methods), yielding a better model than the one based on $\delta^{13}C$ signal. Despite ongoing debate on the relative timing of the aridity and C_4 grass expansion in African habitats, both proxies show a marked increase beginning at 7.2 Ma in our time-binned framework (Extended Data Fig. 2). This suggests that differences in the onset timing of arid regimes and C_4 landscapes are unlikely to explain variation in model fit. Instead, the improvement in fit likely stems from the abrupt rise in dust flux rates at 2.58 Ma, in contrast to the more gradual, sustained increase in the C_4 grasses indicator since 7.2 Ma. As we will see, the Pliocene-Pleistocene transition, 2.58 Ma, marked a pivotal moment in the diversification dynamics of African herbivores."

Page 8 Line 13. "The extent to which aridification primarily tracks global physical trends versus ecosystem-level reorganization is still elusive in our understanding of late Cenozoic African environmental dynamics[12,13,50,57]. Our aridification proxy likely integrates multiple dimensions of environmental change. Beyond capturing increasing dryness, it may reflect shifts in seasonality and hydrological regimes, as well as facets of cascading ecological feedbacks involving vegetation structure, fire regimes, and herbivory. In this sense, it may act as a composite signal of abiotic and biotic restructuring, potentially explaining why it outperforms our proxy for the prevalence of C_4 grasses. It's worth noting in this regard that previous assessments of the impact of late Cenozoic environmental changes on African faunas have yielded conflicting outcomes. In some instances, isotopic values from tooth enamel show lineages adjusting their diets to environmental changes[58–61]. In others, a decoupling of biotic response from environmental change is exemplified by lineages showing dietary conservatism, or in limited changes in the relative local abundance of grazing and browsing taxa[50,59,62].

A key distinction of our approach is its scale, and its emphasis on variation of speciation and extinction rates across lineages and ecomorphs. At this macroevolutionary scale, dietary trends can emerge from differential extinction and speciation rates among lineages, even under widespread conservatism[27,63]. Through such a process of species sorting, major

reconfigurations of African ungulate faunas can take place even under the strongest assumptions of dietary conservatism within ungulate genera, tribes or families.

Future developments in process-based macroevolutionary modeling, such as the framework advanced here, may help reconcile environmental signals operating at basin and regional scales within a unified analytical architecture. Integrating multiple environmental trends into coherent mechanistic models could substantially improve our ability to resolve the spatial heterogeneity of biotic responses across subcontinental regions. Continued refinement of environmental proxies, together with expanded temporal and geographic coverage, will be essential for advancing these integrative approaches and for further clarifying how climatic forcing translated into the uneven macroevolutionary trajectories observed across African ungulate lineages over space and time."

Comment 1.2. I would like to see further discussion and emphasis on the dramatic increase in extinction rates over the past 5 Ma (e.g., in the section entitled "The restructuring of African ungulate faunas"). Looking at the key results figures (e.g., Fig. 1A, Fig. 2, ED Fig 12), the first thing that jumps out is the sharp rise in extinction rates during the Pliocene and Pleistocene for most taxa. The outcome is a net loss of continental-scale diversity over time. This is a really interesting and important result, and one could've written an entire paper about that alone. Though it is not the central question, it's probably worth more attention that it gets in the current manuscript.

Response 1.2 We fully agree with this comment, and we have added a dedicated paragraph addressing this issue in the section *The restructuring of African ungulate faunas*. In the revised analyses, the inferred extinction peak is less pronounced than in the previous version; nevertheless, it remains a prolonged event of sufficient magnitude to warrant explicit discussion. Importantly, in the updated results aridification alone explains a threefold increase in extinction rates, supporting the inclusion of a focused discussion on the macroevolutionary significance of this event.

Page 10 Line 14. "In contrast, the Pleistocene marked a transition to negative diversification across families and ecologies, with speciation rates remaining at Pliocene levels while extinction rates doubled (Fig. 1A). This extinction phase likely represents one of the most impactful biotic crises in Cenozoic times. The early Pleistocene saw an intensification of the high-latitude glacial cycles, which drove profound changes in Africa climate[69] as reflected by a marked increase in our aridification proxy 2.58 Ma (Extended Data Fig. 2). Global temperature fluctuations, increased seasonality in precipitation regimes, and suppressed CO_2 atmospheric concentrations led to substantial ecological change in African ecosystems[13]. This new phase of abiotic forcing also triggered distinct speciation and extinction patterns across lineages and ecomorphs, ultimately shaping the transition from the Miocene–Pliocene phase of elevated diversification to present-day depauperate ecosystems."

Comment 1.3 Throughout the text, the long-term aridification trend is linked to changes in productivity: "long-term aridification trends and the associated changes in productivity",

“likely due to the strong linkage between aridity and primary productivity”, and “prolonged aridification trend and associated declining productivity.” I take no issue with the claim that aridity and productivity are closely linked, but things are a bit complicated from the perspective of large-bodied herbivores. In Africa today, the highest biomass and diversity of ungulates is observed in areas with intermediate rainfall (~600-800 mm/yr; see Fig. 5 and Fig. 6 of Hempson et al 2015). Ungulate diversity and biomass decline at both higher (>800 mm/yr) and lower (<800 mm/yr) rainfall (Hempson et al 2015). This hump-shaped relationship is thought to be a consequence of the interplay between plant biomass, which increases with rainfall, and plant forage quality, which decreases with rainfall (Olff et al 2002). This means that aridification could foster conditions that are beneficial to herbivores (e.g., a decline from 1200 to 800 mm/yr rainfall) or detrimental to herbivores (e.g., a decline from 800 to 400 mm/yr rainfall). What does this complexity mean for the observed relationship between aridity and speciation/extinction rates through time? I’d like to see the authors contend with this in a revised manuscript.

Response 1.3 We thank the reviewer for these insightful remarks. We have expanded the discussion of the links between aridification, changes in precipitation regimes, and the macroevolutionary dynamics of herbivores in the section *Environmental change and ungulate diversification*. In particular, we now provide a more detailed treatment of the bimodal (hump-shaped) relationship between speciation rates in highly hypsodont herbivores and increasing aridity as precipitation regimes shift. We have explicitly aligned this result with the reviewer’s comment by elaborating on the underlying mechanisms and the projected implications of this hump-shaped response, and by directly connecting our interpretation to several of the references suggested by the reviewer.

Page 7 Line 29. “Despite apparently broad HPD intervals, taxa in different hypsodonty categories show disparate responses to aridification in 91% of the posterior samples (see Extended Data Tables). Moderate levels of aridity correlate with increased speciation rates in hypsodont taxa (Fig. 1E). From 11.6 Ma onwards, environmentally-driven speciation accelerated in hypsodont taxa and peaked between 7.2 and 2.58 Ma, coinciding with an accelerated radiation of hypsodont bovids, suids, and elephantids. However, intensifying aridification in Pleistocene times (after 2.58 Ma) resulted in a slowdown in speciation among hypsodont lineages. This hump-shaped relationship between hypsodonty and environmentally-driven speciation (Fig. 1E) likely reflects three phases. First, the onset and first expansion of grass-dominated habitats; second, the attainment of optimal productivity conditions and maximal heterogeneity in woody cover and ecotones at intermediate rainfall[51–53]; and third, a general decline in productivity and a reversal to more homogeneous landscapes under extreme values of our aridity proxy.”

Minor comments R1

Page 2 Line 9: Not until I read the paper did I understand what is meant by “durable dentitions.” Perhaps reword.

OK. We have reworded as “High-crowned, which last longer under hard, abrasive diets”

ED Fig. 7: Brachydont is spelled incorrectly.

Thanks. Fixed.

Page 4 Line 13-14: “its effect on extinction was delayed and milder in larger size categories” The confidence intervals in Fig 1D are quite broad…is this real?

OK. The HPD are quite broad, yet the predictor importance tests provided in Extended Data Tables tell us how often the effect of a trait or interaction of traits is observed across the posterior samples from the MCMC run (column *posterior_prob*). In this particular case, despite overlapping HPD ranges, the combined effect of aridification and body mass shows little overlap within the same samples. We have made this clearer in the text now.

Page 4 Line 34. “Even though HPD intervals in Fig. 1D are relatively broad, extinction responses to aridification are delayed and attenuated in larger size classes in 88% of the posterior samples (see Extended Data Tables).”

Page 5 Line 31: “…yielding a better model than one based on C4 grasses.” I do wonder if this may be partially a consequence of spatial variability in the pattern and timing of C4 expansion across Africa (noted above).

OK. In the new runs obtained with the fixed model, RTT profiles now more clearly track the time-dependent proxies, allowing us to better evaluate their contribution. The section **Environmental change and ungulate diversification** has now been expanded and details why the dust flux proxy outcompetes the alkanes signal:

Page 7 Line 2. “Instead, the improvement in fit likely stems from the abrupt rise in dust flux rates at 2.58 Ma, in contrast to the more gradual, sustained increase in the C_4 grasses indicator since 7.2 Ma. As we will see, the Pliocene-Pleistocene transition, 2.58 Ma, marked a pivotal moment in the diversification dynamics of African herbivores.”

Page 5 Line 37: add comma after “Without the influence of aridification[comma]”

Thanks. Fixed.

Page 6 Line 6-7: “aridification increased the proliferation of hypsodont forms” – can you be clearer about what you mean by proliferation?

OK. This part of the text has been substantially changed in the last version. In the sole instance where we use the word proliferation we specify “species proliferation”.

Page 6 Line 14: what defines “extreme aridification”?

OK. We have reworded this expression, either as “under extreme values of our aridity proxy” or “during the most arid periods”.

Page 6 Line 18: “…with widespread effects on herbivore species” Such as?

OK. This last part of the section has been entirely re-written.

Conclusions: “Our results reveal that this decline was primarily driven by a prolonged aridification trend and associated declining productivity, rather than solely by Late Pleistocene extinctions…” Huh? Wording here is off. How can a long-term loss of megaherbivores over millions of years be driven by Late Pleistocene extinctions?

Thanks. We have rearranged the conclusion paragraph and clarified this and other aspects of the text.

ED Fig 3-5: Maybe it's just me, but I have a real hard time differentiating the blue-greens here. Perhaps an alternate color scheme would be helpful? And the font of some of the labels is tiny.

Thanks. We have changed the palette of these plates and made the legend larger.

Reviewers 2 & 3

Using a sophisticated modeling technique to analyze the diversity dynamics of African fossil ungulates over the last 24 million years, the authors of this study found that large body size did not, in general, increase extinction risks of species, but decreased speciation rates, especially as aridification progressed during the Plio–Pleistocene. As a result, the largest-bodied members of the African megafauna had been in decline millions of years before hominins started to exert severe hunting pressure on them.

The reported major impacts of aridity on mammal dynamics are noteworthy because they seem to paint a divergent picture from some of the recent research on the topic [1–4]. These previous studies found no connection between herbivore paleodiet and aridity changes through the Pliocene-Pleistocene [1], nor changing vegetation with aridity over the past ten million years [4].

This work is significant in its current form as it brings much needed attention back to the speciation side of the history of African mammals—a subject that was central to Vrba’s evolutionary hypothesis [5, 6]. Its impact will be felt by those who study ungulates, east African fauna, and hominin evolution in the context of changing environments around its origin.

We thank Reviewers 2 and 3 for their positive assessment of our study. We are particularly grateful for their recognition of the relevance of our results for understanding the diversification dynamics of African mammals and the role of environmental change in shaping these patterns. We also appreciate their acknowledgement that this work brings renewed attention to the speciation component of African mammal evolutionary history.

Comment 2.1 The significance of this study will be further enhanced if it manages to present a mechanistic synthesis of differing ways in which aridity impacts body size, diet, and diversification. To be fair, the authors do offer some explanatory hypotheses for the observed rate patterns. However, without explicit consideration of alternative hypotheses, those potential explanations give an impression of having been formulated after the fact, even though they are presented as predictions (P. 3, Lines 24–34), and are rather confusing

in some places. For example, as aridification progressed, hypsodont taxa are said to “have encountered more spatially homogeneous resource distributions” (P. 7, Line 5) resulting in their lower extinction rates compared to brachydont taxa, and at the same time, experienced higher speciation rates “under local adaptive pressures”–But wouldn’t homogeneous resource distribution make local adaptive pressures homogenous as well, suppressing speciation? Elaborating on these potential explanations in connection with different modes of speciation (allopatric/peripatric/parapatric) may help make the authors’ arguments more convincing. Overall, we feel that an expanded and clearer discussion of why diversification patterns differed among ecomorphs would make this work more impactful. In addition, a conceptual diagram or a table of proposed mechanisms would help the reader better understand the implications of the results.

Response 2.1 We agree that a more explicit mechanistic synthesis of the multiple correlations and patterns emerging from a model with this level of complexity substantially improves the coherence of the discussion, and in this respect we have followed the recommendations of Reviewers 2 and 3. To integrate and unify this mechanistic perspective throughout the discussion, we now explicitly frame our results within the macroevolutionary concepts of diversity-dependent dynamics, regulated by two main components: on the one hand, the intrinsic speciation and extinction rates of a given group, lineage, or ecomorphology, and on the other hand, the carrying capacity and the crowding effect in the system. Because the BDNN framework allows us to isolate the effects of intrinsic species traits, we can disentangle these intrinsic rates (e.g. Figure 2), while at the same time examining the realized speciation and extinction dynamics reflected in the aggregated rate-through-time patterns produced by the full model (e.g. in Figure 3). Accordingly, throughout the discussion we now consistently address both facets: the intrinsic rate signal associated with the focal ecomorphology at a given time, and the large-scale diversification pattern, which we then interpret in terms of changes in system-level carrying capacity, for example in the context of savanna expansion. We draw conceptually on the framework developed by Marshall and Quental (2016) to articulate this mechanistic synthesis. Although we have not added an additional figure, given the already high density of the existing figures, we believe that the revised discussion is now more streamlined and anchored within a more unified mechanistic context. For example:

Page 9 Line 28. “We suggest that the magnitude of this radiation (with the diversity of some ecomorphs doubling every 1.6 Myrs; Fig. 3) stems from several combined processes. The expanding savannah biome provided access to a much enlarged ecological zone[28,35,36], and promoted habitat heterogeneity, increasing the carrying capacity of the whole system[16]. This effect was multiplied by the higher intrinsic speciation and lower extinction rates of species capable of mixed feeding or grazing (typically meso- and hypsodont taxa; Fig. 1 and Extended data Fig. 7). By allowing the incorporation of lower quality forage, dietary flexibility increases population persistence, thereby reducing extinction risk[32,33] and enhancing the survival of diverging isolates, which promotes speciation[34]. The emergence of novel dietary and social strategies would have relaxed the crowding effect, promoting the coexistence of species in sympatry[27,64]. These innovations included the emergence of migratory habits, which would have

ensured resource access by allowing feeding across multiple seasonal landscapes[23], and of specialized grazers, capable of partitioning roughage types[65]. The combination of these two non-exclusive traits would have promoted speciation through the isolation of populations under local adaptive pressures[66]."

Comment 2.2 Concerning previous research connecting increasing aridity and biotic dynamics of Eastern Africa, the lack of change in enamel isotope values with increasing aridity [1], suggests that mammals may still have been locating their preferred foods (possibly in heterogeneous environments) even in a continental shift to increased aridity. Alternatively, the proxy of aridity (dust flux) may not be a faithful indicator of the vegetation dynamics on the continent. This latter point was at least in part the conclusion of the lack of association in increasing C4 grasslands with increasing aridity [4]. Both of these previous studies highlighted decoupling of aridity from biotic dynamics, in contrast to the findings of the present paper. We encourage the authors to reconcile their findings with those of the previous studies, elaborating on why aridification drove diversity dynamics without major shifts in vegetation or ungulate diet.

Response 2.2 We agree that faunal responses to environmental change are heterogeneous, not only across the ecological strategies of different lineages, but also depending on the scale at which those responses are evaluated. Several dietary studies have shown that some African ungulate groups exhibit a high degree of dietary conservatism through time, with relatively stable isotopic signatures across their evolutionary histories. This pattern reflects within-lineage stability in ecological strategy. However, this represents only one facet of the macroevolutionary change. At a higher level, the key question becomes whether the diversity of lineages maintaining a given strategy proliferate or decline relative to others. Thus, even if browsing or frugivorous lineages remain isotopically conservative through time, they may nonetheless experience net species losses, while mixed-feeding or grass-dominated lineages undergo substantial species proliferation. In such cases, temporal analyses of mean isotopic values within individual groups (e.g. tribes) may show little change, yet a profound restructuring of faunas is still taking place through differential speciation and extinction.

Importantly, the cases highlighted by the reviewers, in which certain taxa remain restricted to specific areas where key resources persist, should be those where differential proliferation is more marked. Here, "resources" should be understood broadly, not only as food, but also as other environmental and ecological factors. These taxa closely match what Elisabeth S. Vrba conceptualized as specialists, which, under her theoretical framework, are precisely the lineages expected to be most strongly affected during periods of environmental disruption. Implicit in this framework is the idea emphasized by the reviewers: remaining restricted to particular resources or conditions is itself the mechanism that, depending on context, increases either extinction risk or speciation rates. These dynamics are inherently context dependent, such that the same degree of niche or resource conservatism may lead to elevated extinction probabilities under some environmental trajectories, or enhanced speciation under others. As such, this perspective is not only fully compatible with the niche and resource conservatism documented in isotopic studies for

some ungulate groups, but is, in fact, a necessary condition under Vrba's theory for specialists to exhibit the higher expected rates of both speciation and extinction in response to environmental change.

More broadly, whereas many of these isotopic studies expect to see anagenetic change within lineages as evidence of ungulate responses to environmental change, our approach addresses macroevolutionary responses as differences in lineage proliferation, that is, evolutionary processes operating above the species level; these represent two distinct and complementary scales at which evolutionary responses can be examined. We have discussed this apparent conflict in the the main text:

Page 8 Line 13. "The extent to which aridification primarily tracks global physical trends versus ecosystem-level reorganization is still elusive in our understanding of late Cenozoic African environmental dynamics[12,13,50,57]. Our aridification proxy likely integrates multiple dimensions of environmental change. Beyond capturing increasing dryness, it may reflect shifts in seasonality and hydrological regimes, as well as facets of cascading ecological feedbacks involving vegetation structure, fire regimes, and herbivory. In this sense, it may act as a composite signal of abiotic and biotic restructuring, potentially explaining why it outperforms our proxy for the prevalence of C_4 grasses. It's worth noting in this regard that previous assessments of the impact of late Cenozoic environmental changes on African faunas have yielded conflicting outcomes. In some instances, isotopic values from tooth enamel show lineages adjusting their diets to environmental changes[58–61]. In others, a decoupling of biotic response from environmental change is exemplified by lineages showing dietary conservatism, or in limited changes in the relative local abundance of grazing and browsing taxa[50,59,62].

A key distinction of our approach is its scale, and its emphasis on variation of speciation and extinction rates across lineages and ecomorphs. At this macroevolutionary scale, dietary trends can emerge from differential extinction and speciation rates among lineages, even under widespread conservatism[27,63]. Through such a process of species sorting, major reconfigurations of African ungulate faunas can take place even under the strongest assumptions of dietary conservatism within ungulate genera, tribes or families."

Comment 2.3 There is one specific conclusion that we think requires additional scrutiny, which is that dietary flexibility suppressed extinction rates (P. 3, Lines 36-37). Extended Figure 7 suggests that hypsodonty and mesodonty are nearly identical in distribution of extinction rates, while only brachydonty appears greater. This suggests that there may be a threshold of crown height that facilitates dietary diversity, but the tallest crown heights do not grant ungulate species additional benefit of increased survival. Likewise, hypsodont and mesodont taxa respond nearly identically to increasing aridity (Fig. 1f). On the other hand, there appears to be a monotonic trend of increasing speciation rate with greater crown height (Extended Figure 7). Could the authors explain why there might be a threshold crown height for extinction but not speciation?

Response 2.3 As noted by the reviewers, Supplementary Fig. 7 shows that the intrinsic effects of mesodonty and hypsodonty are very similar. We have adapted the discussion in the instance where we discuss hypsodonty-related intrinsic rates:

Page 9 Line 32. "This effect was multiplied by the higher intrinsic speciation and lower extinction rates of species capable of mixed feeding or grazing (typically meso- and hypsodont taxa; Fig. 1 and Extended data Fig. 7)."

However, these results reflect isolated effects, as the partial dependence plots capture the effect of each trait state on its own, independent of other predictors. This pattern changes once these character states are considered in interaction with the full set of predictors. For example, when examining Figure 2, clearer structure emerges once hypsodonty is taken into account, with some lineages such as elephantids and suids showing generally lower extinction rates among highly hypsodont and mesodont taxa. While this pattern is not consistently observed across all groups, for instance in bovids, it indicates that the effects of mesodonty and hypsodonty are not uniformly identical across lineages and body-size classes. Accordingly, when lineage-specific patterns are discussed, differences or similarities in patterns in mesodont and hypsodont taxa are addressed on a group-by-group basis, wherever these contrasts are biologically meaningful. For this reason, we do not consider the identification of a specific hypsodonty threshold to warrant a separate discussion. What we do emphasize in the revised text, in line with the reviewers' observations, is that Fig. 1F, similarly to Fig. 1D, shows that the effect of aridification in increasing extinction risk is much more homogeneous across different hypsodonty categories, as it is across different body-size classes. This point is now stated more clearly in the revised manuscript.

Page 8 Line 3. "Aridification is the top predictor of extinction dynamics, accounting for a threefold increase in extinction rates (Fig. 1B). However, unlike its effect on speciation, this impact was more uniformly distributed across the ecospace, showing less variation with respect to body size or hypsodonty levels (Fig. 1 and Extended Data Fig. 3)."

Comment 2.4 Although we appreciate the strength of the 'big data' analyses by the authors, we are concerned by the absence of geographic context in this study, which treats the whole continent of Africa as a single system. We think there are at least two problems that need to be addressed in relation to geography. First, it is unclear to what extent the "speciation" rates estimated in this study pertain to true speciation events in Africa versus immigrations from Eurasia. The authors appear to presume that environmental forces were the key process driving the in-situ speciation, extinction, and diversification of taxa, but there was considerable faunal interchange between Africa and Eurasia during the Neogene [7]. We suggest that the authors mark immigration pulses on the diversity plots. These pulses may be at the African Land Mammal Age (ALMA) boundaries, given that's a primary way in which LMAs are defined. If so, it's unlikely that it will affect interpretation since the authors ran a version of their analysis already using these boundaries, and found it was a worse fit than when using global geological divisions. However, the immigration factor

should still be acknowledged, and how it affects interpretation, if at all, should be discussed.

Response 2.4 The reviewers raise a fundamental issue when analyzing regional diversity patterns, namely that apparent speciation within a focal region may partly reflect dispersal from external regions rather than in situ diversification. This point is directly relevant to our analyses. To assess the magnitude of dispersal effects on our speciation estimates, we have added a new analysis comparing the proportion of first appearance data for species and genera in Africa relative to Eurasia. For each temporal bin, we quantify the fraction of new occurrences in Africa that correspond to species and genera previously sampled in Eurasia. These proportions differ markedly between taxonomic levels: while genus-level proportions are comparatively higher, they drop sharply at the species level, where the contribution of immigrant taxa is extremely low. This analysis allows us to place specific diversification events into clearer context. For example, during the interval between 7.2 and 5.33 Ma, a moment of rapid radiation pulse in bovids and equids, the inferred signal can be associated with the arrival of only three bovid and equid species (three genera), a small contribution relative to the overall magnitude of the diversification event.

Page 9 Line 8. "In bovids and equids, these elevated rates of species proliferation encapsulate dispersal of only three species from Eurasia (Extended Data Fig. 13), suggesting that the diversification signal is not substantially confounded by the influx of immigrant taxa."

Comment 2.5 Second, the highly uneven geographic distribution of major Neogene fossil sites in Africa [3, 8] could, in our opinion, distort some of the diversity patterns reported in this study. For example, had the fossil sampling been much better in west and central Africa--regions that could have provided important refugia for forest-adapted taxa (see e.g., [9])--wouldn't the extinction rates for brachydont taxa be lower? We understand that sampling heterogeneity through time was explicitly modeled in BDNN/PyRate, but whether that is sufficient to overcome sampling heterogeneity across environmentally uneven space is unclear to us. Related to this issue, we think it will be helpful to the reader to see a graphical representation of how much data came from which parts of Africa and which time periods. Overall, we do not think these omissions prohibit publication of this study, but we do think that a revised version of this paper that carefully addresses them will be a more valuable contribution to science.

Response 2.5 We thank the reviewers for drawing our attention to this important point. In the revised version of the manuscript, we have addressed these issues in two complementary ways. First, at the end of the Discussion we have added a paragraph explicitly acknowledging, as noted by previous authors and emphasized by the reviewers, that the spatial distribution of the Neogene–Quaternary African fossil record is not homogeneous, as expected. We further clarify that the localities included in this study, which also form the basis of most African faunal analyses, capture trends across a range of environments spanning from tropical deciduous forests to savannas, including woodlands as well as different types of grasslands and wetlands. By contrast, humid tropical

rainforests are comparatively underrepresented in the fossil record, and we therefore explicitly state that our results do not directly extend to such environments.

Page 11 Line 13. "The macroevolutionary processes described here must be interpreted within the spatial and environmental limits of the African fossil record[13] (Extended Data Fig. 14). Neogene and Quaternary African sites primarily capture a broad range of savanna environments, including woodlands, wetlands, and grasslands. It is mainly within this environmental and spatial context that patterns of African megaherbivore decline had been previously documented[10,11,18], and within which our findings should be understood. The processes described here are not straightforwardly extrapolated to tropical forests[73], where brachydont taxa likely were buffered against extinction rates, and where grazers did not reach such high levels of diversity[68,71]."

In addition, we have included a new figure, Extended Data Figure 14, referenced in this paragraph, which illustrates the temporal prevalence of different African geographic regions in the sampling through time.

Comment 2.6 The methods are thoroughly described, and we greatly appreciate the inclusion of the commands and outputs of the BDNN model, almost to a tutorial degree, which fills in details that do not currently exist on the developers website or in other documentations. Nevertheless, we were unable to check the reproducibility of the study using the provided R script because of this function, which appeared custom-made:

separa_caracteres()

We encourage the authors to (1) provide the function so that others can reproduce the results and even cite the code for inspiration in their own analyses and (2) check again that the rest of the script is capable of being run by someone downloading it as supplemental material.

Response 2.5 We have taken note of this issue in the script, namely the absence of the function now referred to as *split_characters* in the original version. We have thoroughly revised the code to ensure that it runs correctly and independently of the session in which the main analyses for the manuscript were conducted. We sincerely thank the reviewers for taking the time to check the code in detail, fully recognizing that this is a demanding task, and we are grateful for their rigor and careful attention in this regard.

In what follows, we provide a point-by-point account of the revisions that we consider most significant or that warrant specific attention. Reviewers' comments are shown in black and our responses are in green.

Reviewer 1

I appreciate the opportunity to examine the revised version of this manuscript. Overall, I believe that the authors have made a good effort to address the reviewer concerns, resulting in a stronger paper. The revisions are appropriate, and the few rebuttals are quite reasonable. I have mostly minor edits to offer on this draft:

We sincerely appreciate the time and effort invested by the reviewer in evaluating our manuscript. We firmly believe that the comments and suggestions provided have been invaluable in strengthening the work, and that the revised manuscript is now considerably more robust than its original version.

Comment 1.1. Here you argue that aridity proxy may outperform the C4 grass proxy because the former may capture other important ecological feedbacks (e.g., veg structure, fire, herbivory). But might the same also be true of the C4 grass proxy? Almost certainly the expansion of C4 grasses is associated with changes in fire regimes, herbivory, etc (and likely also encompasses some aspects of climate too).

Response 1.1 OK. We have rephrased the paragraph and incorporated this shared nature of the two proxies, while improving the connectivity with the following paragraph.

"Our aridity and C4 grass proxies likely reflect different mechanistic facets of the same broad transformation of African ecosystems since the Middle Miocene, spanning shifts in seasonality and hydrological regimes to cascading ecological feedbacks involving vegetation structure and fire regimes. Because herbivory both responds to and modulates these trends[58], faunal dynamics are themselves embedded within the environmental feedback system. In this regard, our results suggest that herbivore turnover is more tightly coupled to the dimension of environmental change captured by moisture availability than to shifts in vegetation composition.

That disentangling these coupled processes is far from straightforward is illustrated by the conflicting biotic responses reported in the literature. [...]"

Comment 1.2. Line 410-412: I still don't follow this sentence: "This scenario contrasts with hypotheses attributing megaherbivore decline primarily to Late Pleistocene extinctions, which, as we show, lacked size or dietary selectivity" A long-term decline of megaherbivores cannot be a attributed to something that happened within the last ~100,000 years. Do you mean "…decline to the same mechanisms responsible for Late Pleistocene extinctions…"?

Response 1.2 OK. We have rephrased, better conveying our intended meaning: that classical interpretations of megaherbivore decline have treated it as a shorter and more recent phenomenon, confined to the latest Pleistocene.

"This scenario contrasts with notions of an abrupt megaherbivore decline attributed primarily to Late Pleistocene extinctions, which, as we show, lacked size or dietary selectivity."

Reviewers 2 & 3

We thank the authors for their careful consideration of our comments and suggestions, at times even going beyond the recommendations we outlined. Overall, we find substantial improvement to this manuscript with changes that will increase its impact and accessibility to readers.

We would like to thank both reviewers for the time and effort dedicated to evaluating our manuscript, as well as for conducting such a well-coordinated review. All comments provided have been highly constructive and have contributed to significantly strengthening the manuscript.

Comment 2.1 Line 213: If we understand correctly, Reviewer 1 was concerned about spatial mismatch between the faunal data and the paleoenvironmental proxy data, rather than spatial averaging of the latter; i.e., the potential problem is more about bias than scale. For the sake of transparency, it will be good to state (as Reviewer 1 pointed out) where geographically the proxy data come from and discuss to what extent the authors think the proxy datasets reflect continental trends.

Response 2.1 OK. We have added information on the origin of the sea cores in the supplementary text.

Comment 2.2 Extended Data Fig. 13, and the manuscript text assessing the impact of Eurasian immigrant taxa on African diversification dynamics is an exciting addition, though we were confused as to aspects of interpreting the figure. We feel that neither the figure caption nor the manuscript text provides adequate context as to what the "Immigration" and "FADs" data points represent. The authors' response to our initial comments states "...for each temporal bin, we quantify the fraction of new occurrences in Africa that correspond to species and genera previously sampled in Eurasia." Combined with a text example of time bin 7.2 to 5.33 Ma having three species immigrating from Eurasia, this should correspond to the blue bar "FADs" data. What then is the "Immigration" data? Is it overall first appearances in Africa during that time bin? If so, shouldn't these labels be the other way around?

Response 2.2. The reviewers are correct, the color labels in the legend were misplaced. We have changed the figure accordingly.

Comment 2.3 Please also provide more information on how immigrants from Eurasia to Africa (as opposed to those from Africa to Eurasia) were identified. For example, does "previously sampled in Eurasia" mean occurrence in an earlier time bin? If so, taxon name-matching may, once again, underestimate immigration events when individual time bins have long spans, so that should be acknowledged.

Response 2.3. We have clarified in the manuscript how we estimate an earlier Eurasian FAD. Our approach is not based on time bins but on the upper age range of individual occurrences. When the FAD in Eurasia predates that in Africa by at least 0.2 Myr, we consider the taxon to have a Eurasian origin. This is a deliberately conservative threshold, meaning that our approach is biased towards recognizing Eurasian origins rather than inflating African ones.

“To account for the role of dispersal in shaping speciation rates among African ungulates, we estimated first appearance dates (FADs) of species and genera both in Africa and in Eurasia. Then we compared the ages of these FADs, and considered that a taxon had an Eurasian origin if the Eurasian FAD was at least 0.2 Myr older than the African LAD.”

We have added a similar explanatory text in the caption of Extended Data Fig. 13.

Comment 2.4 We are concerned that the species-level name-matching between Eurasia and Africa may substantially underestimate the true frequency of immigration events. Unless the temporal density of the fossil dataset is very high, species name-matching is unlikely to capture many of the immigrant Eurasian taxa when they first appear in Africa, since there is a sensible expectation that geologically rapid local adaptation of immigrants will result in paleontologists recognizing them as new species, even though they are part of the same lineages that came from Eurasia. Our suggestion is to avoid this issue by focusing more on the genus-level data that the authors have already included, with additional contextual information for the reader in the form of figure caption or manuscript text as described above.

Response 2.4. We thank the reviewers for raising this point. A reference documenting the dispersal of three bovid and equid species between 7.2–5.33 Ma has been added. Both analytical scales were incorporated as part of the revised version to ensure readers have the context needed to interpret results independently. Since the study is explicitly framed around speciation and extinction, species-level dispersal discussions are maintained as the primary framework.
