# Supplementary material for: Lower speciation, not higher extinction, drove African megaherbivore diversity collapse
Source: Nat Commun. 2026 Jul 31;17:9373. doi: 10.1038/s41467-026-76105-2 (PMC13534676; doi:10.1038/s41467-026-76105-2)
Supplement: Supplementary file 4 [file 41467_2026_76105_MOESM4_ESM.pdf]

Description of Additional Supplementary Files

Supplementary Data 1

Description:

The Excel file *Supplementary Data 1* contains data used to run the analyses, as well as the main analytical outputs. The file comprises seven sheets, each with specific content:

- **Occurrence Data:** includes the 3,327 records of African ungulate occurrences considered in this study.
- **Trait Data:** provides body size and hypsodonty classifications, along with three scaled eigenvectors derived from the family-level phylogeny, for the 396 species.
- **Environmental Proxies:** reports scaled values of the environmental variables for each temporal bin, along with the original mean and standard deviation of each variable, which can be used to rescale axes when generating Partial Dependence Plots (PDP).
- **Rate Shifts:** lists the time points used for temporal shifts in the diversification rates.
- **Sampling Shifts:** details the time points associated with changes in sampling rates.
- **RTTs:** contains time-series estimates of diversification rates for each combination of the two traits, both aggregated for all lineages and separately for each family across temporal bins. These data correspond to Figure 3 in the main text.
- **RTT without aridity:** presents the same results as the previous sheet, but excluding the effect of aridity from the predictions. These data were used to generate Supplementary Fig. 8.
- **Predictors Importance Speciation Dust:** contains results from the analysis of predictor importance and their interactions (-BDNN_pred_importance - BDNN_pred_importance_interaction) in relation to speciation rates, based on the model where dust flux is the environmental proxy. Both Delta Likelihood values and SHAP values are highlighted.
- **Predictors Importance Extinction Dust:** contains results from the analysis of predictor importance and their interactions in relation to extinction rates, based on the model where dust flux is the environmental proxy. Both Delta Likelihood values and SHAP values are highlighted.
- **Predictors Importance Speciation C_{35}:** contains results from the analysis of predictor importance and their interactions in relation to speciation rates, based on the model where the $\delta^{13}C$ isotopic signal in long chain n-alkanes (C_{35}) is the environmental proxy. Both Delta Likelihood values and SHAP values are highlighted.
- **Predictors Importance Extinction C_{35}:** contains results from the analysis of predictor importance and their interactions in relation to extinction rates, based on the model where the $\delta^{13}C$ isotopic signal in long chain n-alkanes (C_{35}) is the environmental proxy. Both Delta Likelihood values and SHAP values are highlighted.
